# Multiple knockout mouse models reveal lincRNAs are required for life and brain development

**DOI:** 10.7554/eLife.01749

**Published:** 2013-12-31

**Authors:** Martin Sauvageau, Loyal A Goff, Simona Lodato, Boyan Bonev, Abigail F Groff, Chiara Gerhardinger, Diana B Sanchez-Gomez, Ezgi Hacisuleyman, Eric Li, Matthew Spence, Stephen C Liapis, William Mallard, Michael Morse, Mavis R Swerdel, Michael F D’Ecclessis, Jennifer C Moore, Venus Lai, Guochun Gong, George D Yancopoulos, David Frendewey, Manolis Kellis, Ronald P Hart, David M Valenzuela, Paola Arlotta, John L Rinn

**Affiliations:** 1 Department of Stem Cell and Regenerative Biology, Harvard University, Cambridge, United States; 2 Broad Institute of MIT and Harvard, Cambridge, United States; 3 Computer Science and Artificial Intelligence Laboratory, Massachusetts Institute of Technology, Cambridge, United States; 4 Department of Cell Biology and Neuroscience, Rutgers, The State University of New Jersey, New Brunswick, United States; 5 Department of Genetics, Rutgers, The State University of New Jersey, New Brunswick, United States; 6 VelociGene, Regeneron Pharmaceuticals Inc., Tarrytown, United States; 7 Department of Pathology, Beth Israel Deaconess Medical Center, Harvard Medical School, Boston, United States; Howard Hughes Medical Institute, New York University School of Medicine, United States

**Keywords:** long noncoding RNAs, knockout mouse models, lethality, developmental defect, brain development, Mouse

## Abstract

Many studies are uncovering functional roles for long noncoding RNAs (lncRNAs), yet few have been tested for in vivo relevance through genetic ablation in animal models. To investigate the functional relevance of lncRNAs in various physiological conditions, we have developed a collection of 18 lncRNA knockout strains in which the locus is maintained transcriptionally active. Initial characterization revealed peri- and postnatal lethal phenotypes in three mutant strains (*Fendrr*, *Peril*, and *Mdgt*), the latter two exhibiting incomplete penetrance and growth defects in survivors. We also report growth defects for two additional mutant strains (*linc–Brn1b* and *linc–Pint*). Further analysis revealed defects in lung, gastrointestinal tract, and heart in *Fendrr*^*−/−*^ neonates, whereas *linc–Brn1b*^*−/−*^ mutants displayed distinct abnormalities in the generation of upper layer II–IV neurons in the neocortex. This study demonstrates that lncRNAs play critical roles in vivo and provides a framework and impetus for future larger-scale functional investigation into the roles of lncRNA molecules.

**DOI:** http://dx.doi.org/10.7554/eLife.01749.001

## eLife digest

The mammalian genome is comprised of DNA sequences that contain the templates for proteins, and other DNA sequences that do not code for proteins. The coding DNA sequences are transcribed to make messenger RNA molecules, which are then translated to make proteins. Researchers have known for many years that some of the noncoding DNA sequences are also transcribed to make other types of RNA molecules, such as transfer and ribosomal RNA. However, the true breadth and diversity of the roles played by these other RNA molecules have only recently begun to be fully appreciated.

Mammalian genomes contain thousands of noncoding DNA sequences that are transcribed. Recent in vitro studies suggest that the resulting long noncoding RNA molecules can act as regulators of transcription, translation, and cell cycle. In vitro studies also suggest that these long noncoding RNA molecules may play a role in mammalian development and disease. Yet few in vivo studies have been performed to support or confirm such hypotheses.

Now Sauvageau et al. have developed several lines of knockout mice to investigate a subset of noncoding RNA molecules known as long intergenic noncoding RNAs (lincRNAs). These experiments reveal that lincRNAs have a strong influence on the overall viability of mice, and also on a number of developmental processes, including the development of lungs and the cerebral cortex.

Given that the vast majority of the human genome is transcribed, the mouse models developed by Sauvageau et al. represent an important step in determining the physiological relevance, on a genetic level, of the noncoding portion of the genome in vivo.

**DOI:** http://dx.doi.org/10.7554/eLife.01749.002

## Introduction

Mammalian genomes encode thousands of long noncoding RNAs (lncRNAs), which are emerging as key regulators of cellular processes (Rinn and Chang, 2012; Mercer and Mattick, 2013). Gain- and loss-of-function approaches in cell-based in vitro systems have been useful in uncovering important roles for lncRNAs, such as modulating chromatin states, maintaining cellular identity (i.e. pluripotency) and regulating cell cycle and translation (Tsai et al., 2010; Guttman et al., 2011; Wang et al., 2011; Yoon et al., 2012). Genome-wide association and expression profiling studies in humans have also identified correlations between lncRNA mutation, misregulation and disease states (Visel et al., 2010; Cabili et al., 2011; Brunner et al., 2012). Yet, direct in vivo, genetic evidence of the functional significance of lncRNAs as a class of transcripts remains elusive.

Functional studies in knockout mouse models have provided compelling evidence for the requirement and sufficiency of particular transcripts for organ development and function. However, small and large-scale efforts have focused primarily on protein coding genes, leaving long noncoding RNAs vastly understudied (Edwards et al., 2011; White et al., 2013). Of the few lncRNA mutant mice generated to date, most involved previously studied and classic examples of lncRNAs (e.g., *Xist*, *H19*, *Kcn11ot1*, *Malat1*) (Anguera et al., 2011; Gomez et al., 2013; Gordon et al., 2010; Moseley et al., 2006; Nakagawa et al., 2011; Ripoche et al., 1997; Zhang et al., 2012), leaving the more recent large-scale RNA-Seq-derived catalogs of lincRNAs largely unexplored. The progress made in these early studies to initiate functional characterization of lncRNAs in vivo now warrants a larger investigation of their biological importance to development and disease.

To address this directly, and to begin to explore the roles of lncRNAs in vivo, we have established a novel cohort of lncRNA knockout mice. We focused on a subgroup of lncRNAs called long intergenic noncoding RNAs (lincRNAs), such that genetic deletion would not overlap known protein coding genes or other known gene annotations. We have implemented a generalized and logical lincRNA candidate selection process that leverages a collection of cell-based functional assays, RNA-sequencing data and computational analyses. This approach led us to identify 18 lincRNAs for targeted deletion in mouse. Initial characterization of these new knockout strains demonstrated key functional roles in viability, development of the cerebral cortex and other developmental processes. In this study, we describe the observed phenotypes for five strains within this collection. Collectively, these data provide evidence that lincRNAs play central roles in mammalian development and physiology.

## Results

### Global characterization of lincRNA knockout candidate selection

**Figure 1.**
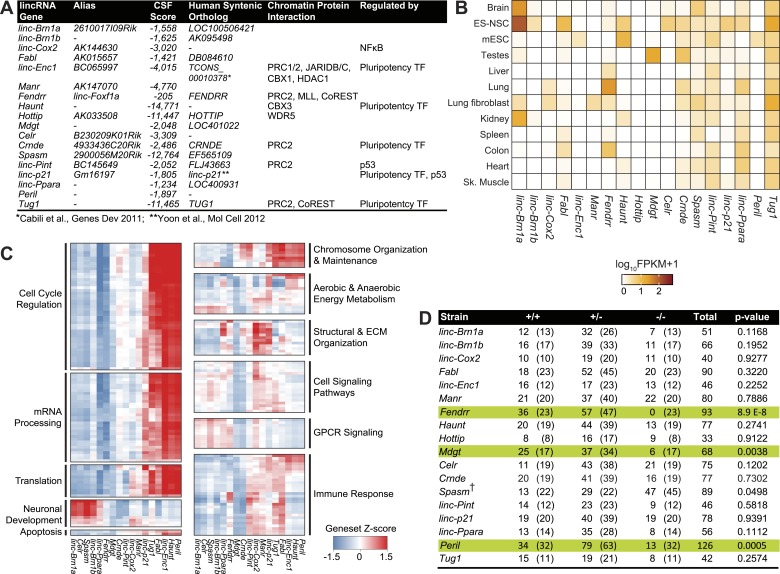
Properties of the 18 lincRNA candidates and Mendelian inheritance. (**A**) List of the 18 lincRNA candidates for targeted deletion in mouse and overview of criteria used for their selection. (**B**) Heatmap of log_10_ FPKM+1 expression levels of the 18 lincRNAs in a panel of adult mouse tissues and cell lines via RNA-Seq. (**C**) Guilt-by-association (GBA) analysis for 17/18 lincRNA candidates. Individual tiles represent significant (p<1.0 × 10^−6^) gene sets from the CP Reactome collection at MSigDB. Tiles are filled based on the Z-score of the Pearson correlation values between a given lincRNA and the genes within the gene set across a compendium of RNA-Seq samples. (**D**) Mendelian inheritance of the 18 lincRNA mutant alleles from the progeny of heterozygote intercrosses. Numbers of observed and expected (in parenthesis) wild-type (+/+), heterozygote (+/−) and homozygote (−/−) mice are indicated. Mice were genotyped at weaning age. The p value is based on X2 test. † The *Spasm* gene is located on the X chromosome. **DOI:** http://dx.doi.org/10.7554/eLife.01749.003

To more globally understand the physiological significance of lincRNAs, we combined a step-wise lincRNA selection pipeline with a genetic approach to engineer a cohort of lincRNA knockout mouse strains for functional analysis. We integrated several computational and experimental data sets to select *bona fide* lincRNA candidates across three lincRNA catalogs (Guttman et al., 2009; UCSC and Refseq). First, all transcripts with identifiable Pfam domains or those overlapping known non-lncRNA annotations (e.g., annotated protein coding genes, microRNAs, tRNAs and pseudogenes) were excluded. Second, we excluded any remaining transcripts with conserved protein coding potential, including the potential to produce small peptides, by performing stringent codon-substitution frequency (CSF) analysis (Lin et al., 2011). We previously demonstrated that this algorithm is capable of discriminating known functional small peptides down to 11 amino acids (Guttman and Rinn, 2012; Guttman et al., 2013). Using selective criteria, we restricted candidate lincRNAs to those with CSF scores <−200 (ranging from −205 to −14,771, Figure 1A). Then, we examined existing ribosome profiling datasets to quantitate the ability of these transcripts to engage the ribosome (Figure 1—figure supplement 1). None of the tested candidates were shown to have clear translation efficiency or codon bias according to proposed standards (Guttman et al., 2013). Finally, through genome lift-over of our mouse lincRNAs to the human genome (build hg19), we examined mass spectrometry data by Gascoigne et al. (2012) to discard transcripts with mapped peptides. We kept transcripts with only two or less mass spectrometry tags, consistent with low levels of background ribosome association observed by Guttman et al. (2013) (Supplementary file 1B) . Thus, based on an exhaustive analysis of existing annotations, standard evolutionary and ribosome profiling metrics, as well as mass spectrometry data, these candidates do not appear to contain protein coding potential.

**Figure 1—figure supplement 1.**
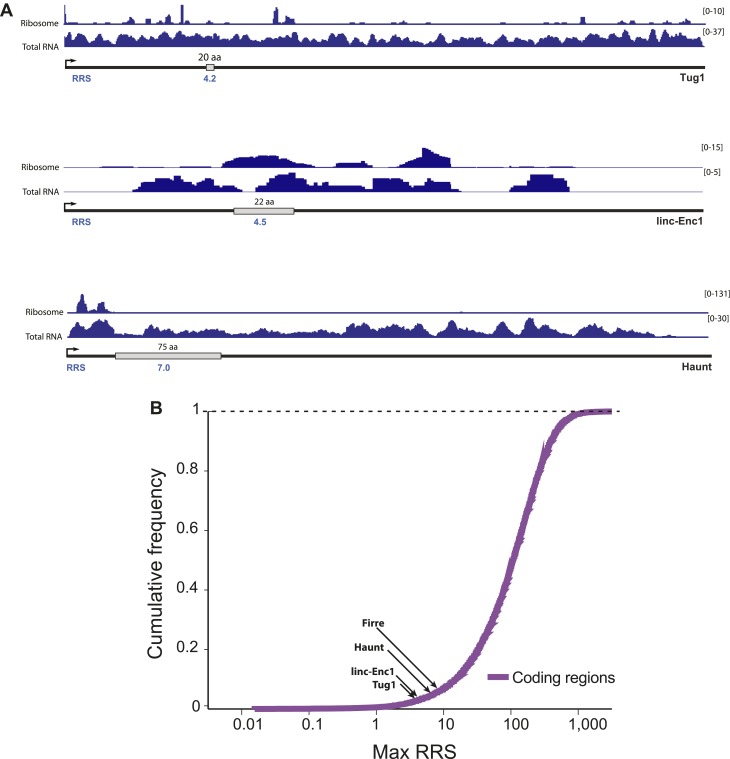
Ribosome release score of lncRNAs. The ribosome release score (RRS) measures evidence of ribosome disassociation at the stop codon of a putative coding region. A putative coding region is defined as the region between a start codon and then next in-frame stop codon. Its putative 3′-UTR is defined as the region beginning immediately after the stop codon and ending at the next start codon in any frame. The RRS score is defined as the total number of ribosome-associated reads overlapping the putative coding region, divided by the number of ribosome-associated reads contained within the 3′-UTR. This ratio is then normalized by the same ratio for total RNA reads. (**A**) The ORF with maximum RRS score for each lincRNA is shown. (**B**) Maximum RRS scores of lincRNAs are compared to the distribution of RRS scores for known coding genes. **DOI:** http://dx.doi.org/10.7554/eLife.01749.004

**Figure 1—figure supplement 2.**
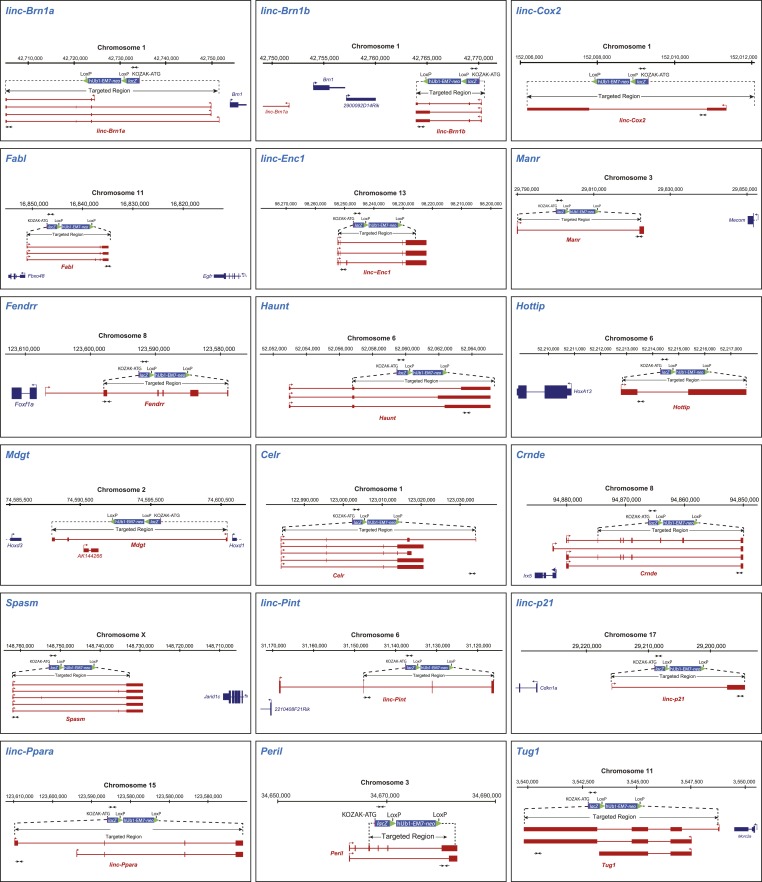
lincRNA candidates knockout targeting strategy. Genomic locus and targeted deletion scheme for the 18 lincRNA candidates. Briefly, each lincRNA gene was replaced by a ß-galactosidase (*lacZ*) reporter cassette containing a KOZAK-ATG sequence, polyadenylation signal, and a LoxP-flanked neomycin (*neo*) resistance gene driven by the human ubiquitin C promoter (hUb1) (mammalian cells) and EM7 promoter (for gap repair cloning selection in bacteria). Arrows indicate location of the primer sets used for genotyping. **DOI:** http://dx.doi.org/10.7554/eLife.01749.005

From this stringent list of lincRNAs, we selected 18 candidates by further considering additional criteria: (1) Presence of an observed orthologous human lincRNA with syntenic conservation as determined by TransMap (Zhu et al., 2007); (2) Presence of canonical chromatin features of actively transcribed genes such as histone H3 lysine 4 trimethylation (H3K4me3) at the promoter and histone H3 lysine 36 trimethylation throughout the gene body (Guttman et al., 2009, 2010); (3) Local ‘enhancer-like’ signatures based on published P300, RNA polymerase II, histone lysine 4 monomethylation (H3K4me1) and histone lysine 27 acetylation (H3K27ac) chromatin states (Shen et al., 2012). We selected 6/18 candidates lncRNA loci that have putative ‘enhancer’ modifications, thus allowing to investigate the newfound roles of lncRNAs in ‘enhancer’ activity (Ørom et al., 2010; Wang et al., 2011). Collectively, this step-wise selection process resulted in 18 high-quality candidate lincRNAs with diverse features, for targeted deletion and phenotypic characterization (Figure 1A, Supplementary file 1A for coordinates and nomenclature details).

We first examined the gene-expression patterns of the candidate lincRNAs using RNA-sequencing of various adult tissues and cell types. Several lincRNAs presented more restricted patterns of expression, suggesting strong tissue specificity, for example *Celr in* embryonic stem (ES)-derived neural stem cells (NSCs)*, linc–Enc1* in mouse ES cells, *Manr* and *linc–Cox2* in lung, whereas a select few showed more ubiquitous expression across tissues (*linc–Pint*, *Spasm*, *linc–Ppara, Tug1*) (Figure 1B). Most of the candidate lincRNAs (12/18) were expressed in the adult brain or in ES-derived NSCs (Figure 1B).

Since most candidate lincRNAs in this screen have unknown biological roles, we leveraged our RNA-sequencing data using guilt-by-association (GBA) analysis to generate hypotheses on functional significance by comparing the expression of each lincRNAs to protein coding genes of known function (‘Materials and methods’) (Figure 1C). GBA predicted lincRNA activities across a wide range of pathways and biological processes, ranging from regulation of the cell cycle and chromosome organization and maintenance, to neuronal differentiation and immune response (Figure 1C). Overall, these analyses suggested diverse potential roles for our candidate lincRNAs.

### Genetic analysis of lincRNA knockout mice reveals key roles in mammalian development

To study the function of the selected 18 lincRNAs in vivo and to resolve, at the cellular level, their expression pattern in different organs, we generated knockout mouse strains for each candidate by replacing the lincRNA gene with a *lac*Z reporter cassette (‘Materials and methods’, Figure 1—figure supplement 2 for details) (Valenzuela et al., 2003). These new strains, which are the first lincRNA knockout models to incorporate a reporter, more than double the number of models available for investigation and constitute an important resource that will be used to better understand the functional contribution of lincRNAs to mammalian biology.

To assess the requirement for each lincRNA in embryonic development and viability, we examined the progeny from heterozygote intercrosses for all 18 strains. Genotyping of weanlings (21 days old) revealed normal Mendelian segregation of mutant alleles in 15 of the 18 strains (Figure 1D). For the three remaining strains *Peril, Mdgt* and *Fendrr,* the progeny of heterozygote intercrosses contained much lower numbers of homozygote mutants than expected. Only 13 *Peril*^*−/−*^ mice (of an expected 32), and 6 *Mdgt*^*−/−*^ mice (of an expected 17) were found at weaning age, indicating that deletion of *Peril* and *Mdgt* leads to reduced viability with >50% and 65% penetrance, respectively (Figure 1D). Closer examination of *Mdgt* pups revealed that homozygous mutants died within 2 weeks after birth. For *Fendrr,* no homozygous mutants were found at weaning age (Figure 1D), indicating that the lethal phenotype for this strain is fully penetrant. Thus, 3 out of the 18 (17%) lincRNA knockout strains generated have a lethal phenotype, confirming that specific lincRNA genes are required for viability.

### *Peril*^*−/−*^ mice have reduced viability and die shortly after birth

**Figure 2.**
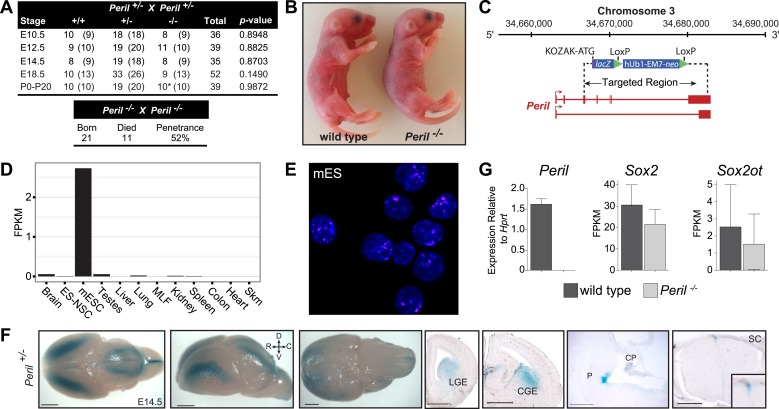
Deletion of *Peril* leads to reduced viability. (**A**) Genotyping results from heterozygote intercrosses (Upper panel) at different developmental stages (*pups dying within days after birth) and homozygote intercrosses (Lower panel) at birth. The p value is based on X^2^ test. (**B**) Newborn (P0) *Peril*^*−/−*^ mutants and wild-type littermates. (**C**) *Peril* genomic locus and targeting scheme. (**D**) RNA-Seq expression profile for *Peril* across a panel of mouse tissues and cell types. (**E**) Single-molecule FISH targeting *Peril* in wild type mouse embryonic stem cells (mES). Nuclei were stained with DAPI. (**F**) Whole mount and coronal section *lacZ* stainings reporting expression of *Peril* in the brain and spinal cord of a heterozygote E14.5 embryo. LGE/CGE, Lateral and Caudal Ganglionic Eminence; P, Pons; CP, Choroid Plexus; SC, Spinal Cord; D, dorsal; V, ventral; R, rostral; C, caudal. Scale bars = 1 mm, whole brains; 500 µm, sections. (**G**) Relative expression levels of *Peril* as assessed by RT-PCR and expression estimates (FPKM) of the neighboring genes *Sox2* and *Sox2ot* as assessed by RNA-Seq in E18.5 brain of homozygote mutant and wild-type littermates (n = 2 each). **DOI:** http://dx.doi.org/10.7554/eLife.01749.006

To determine the onset of *Peril*^*−/−*^ mice lethality, we monitored survival at both early and late stages of embryonic development. Since normal Mendelian ratios were observed up to E18.5 (Figure 2A), and pups appeared macroscopically normal at birth (Figure 2B), we monitored newborns to see if lethality occurred perinatally. We observed that 50% (5/10) of *Peril*^*−/−*^ pups died within 2–20 days after birth (Figure 2A). A similar percentage (52%, 11/21) of newborn deaths occurred in the progeny of intercrosses from surviving *Peril*^*−/−*^ mice (Figure 2A, lower panel). These results confirmed the reduced viability of *Peril* mutants (∼50% penetrance) with pups dying early after birth.

**Figure 2—figure supplement 1.**
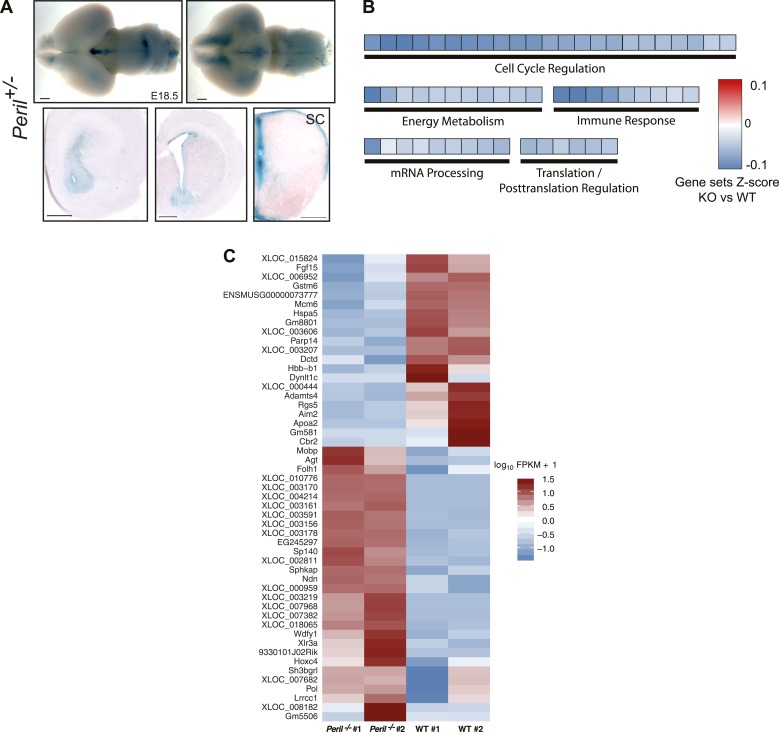
*Peril* E18.5 brain differential RNA-Seq analysis. (**A**) *lacZ* staining reporting expression of *Peril* in brain and spinal cord of a heterozygote E18.5 embryo. SC, Spinal Cord. Scale bars = 1 mm whole brains, 500 µm sections. Scale bars = 1 mm whole brains, 500 µm sections. SC, spinal cord. (**B**) GSEA of *Peril*^*−/−*^ vs wild-type E18.5 total brain RNA-Seq. Each tile is a significant (q<0.001; Mann-Whitney, BH) gene set from the Reactome collection at mSigDB, based on the *Peril*^*−/−*^/wild-type ranking of test-statistic values from a Cuffdiff2 differential analysis. Tiles are shaded based on the z-score of the test-statistic for genes within the given gene set, relative to all genes for a given condition to show direction of expression relative to wild-type. (**C**) Heatmap of significant (q<0.05, CuffDiff2) differentially expressed genes (log_10_ FPKM+1) in *Peril*^*−/−*^ vs wild-type E18.5 total brain by RNA-Seq. **DOI:** http://dx.doi.org/10.7554/eLife.01749.007

*Peril* is a seven exon transcript derived from a 18.2 Kb genomic locus located ∼110 Kb downstream of the pluripotency factor *Sox2* (Figure 2C). Using mouse ES cell (mES) cDNA, we were able to clone two distinct isoforms (1,831 bp and 631 bp) (Figure 2C). RNAseq expression profiling from a panel of mouse tissues and cell lines showed that *Peril* was highly enriched in mouse ES cells, but also expressed at lower levels in adult brain and testes (Figure 2D). The *Peril* transcript is predominantly nuclear as revealed by RNA fluorescence in situ hybridization (FISH) in mouse ES cells (Figure 2E). Using the knocked-in *lacZ* reporter in heterozygote embryos, tight temporal and spatial regulation of *Peril* was found in the brain and spinal cord of E14.5 and E18.5 embryos (Figure 2F, Figure 2—figure supplement 1A).

To identify putative pathways affected by deletion of *Peril*, we performed RNA-sequencing on E18.5 brains from *Peril*^*−/−*^ and wild-type littermates (n = 2). Analysis of *Peril* expression levels confirmed the deletion of *Peril* (Figure 2G). Expression of the pluripotency factor *Sox2* and its overlapping noncoding RNA *Sox2ot* were not significantly affected in the knockout brains (Figure 2G). Differentially regulated genes and gene set enrichment analysis (GSEA) revealed downregulation of genes involved in cell cycle regulation, energy metabolism, immune response, and mRNA and protein processing in *Peril*^*−/−*^ brains relative to wild-type controls (Figure 2—figure supplement 1B and C). This indicates that the transcriptional programs within the brain are in fact affected by deletion of *Peril.* Yet, it remains unclear if these changes underlie the observed lethality.

### *Fendrr*^*−/−*^ is perinatal lethal due to defects in multiple organs

**Figure 3.**
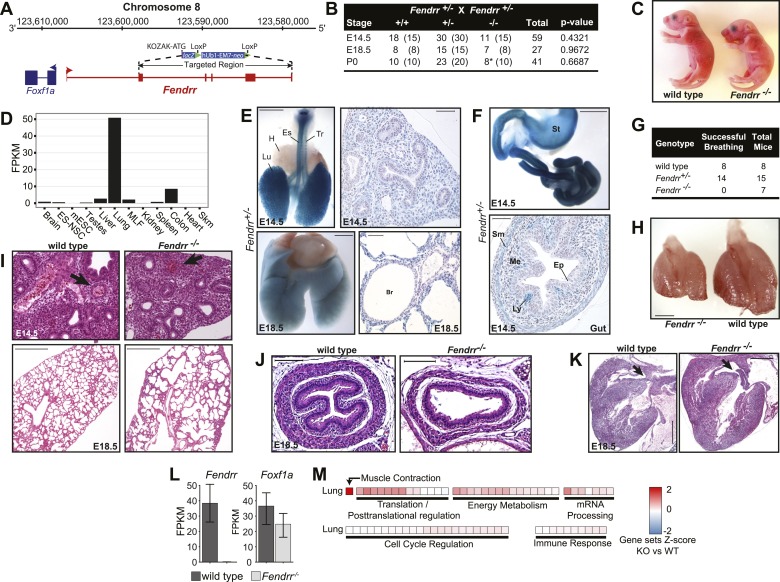
*Fendrr*^*−/−*^ pups have multiple defects in lung, heart and gastrointestinal tract. (**A**) *Fendrr* locus and targeting strategy. Arrows indicate location of the primers used for genotyping. (**B**) Genotyping results from heterozygote intercrosses at embryonic stages E14.5, E18.5 and at birth (P0). The p value is based on X^2^ test. (*) All newborns died within 24 hr after birth. (**C**) *Fendrr*^*−/−*^ E18.5 embryos and wild-type littermates. (**D**) RNA-Seq expression profile for *Fendrr* across a panel of mouse tissues and cell types. (**E** and **F**) *lacZ* reporter stained organs and sections showing expression of *Fendrr* in specific regions of the lung (Lu), trachea (Tr) and esophagus (Es), but not in heart (**H**) in E14.5 and E18.5 embryo (**E**) and in the gut and stomach (St) (**F**). Sm, smooth muscle; Ep, Epithelia; Me, Mesenchyme; Ly, Lymphoid aggregates. Scale bars = 1 mm whole organ, 200 µm sections. (**G**) Number of E18.5 embryos successfully breathing after surgical delivery. (**H**) Size difference of *Fendrr*^*−/−*^ lungs at E14.5 compared to wild-type littermates (n = 3 each). (**I**–**K**) Representative hematoxylin and eosin (H&E) stained sections showing unstructured vessels (arrow) in E14.5 *Fendrr* mutant lungs compared to wild type littermates (n = 3) (**I**, upper panels), alveolar defects at E18.5 (**I**, lower panel), thinner mesenchymal layer of the mucosa and external smooth muscle layer of the oesophagus (**J**) and ventricular septal defects in the heart (**K**) of *Fendrr*^*−/−*^ E18.5 embryos compared to wild type (n = 3). Scale bars= 500 µm, 100 µm for esophagus. (**L**) RNA-Seq expression levels of *Fendrr* and the neighboring coding gene *Foxf1a* in E18.5 lung of homozygote mutant and wild-type littermates (n = 2 each). (**M**) GSEA of *Fendrr*^*−/−*^ vs wild-type E18.5 lung and total brain RNA. Each tile is a significant (q<0.001; Mann-Whitney, BH) gene set from the Reactome collection at mSigDB, based on the *Fendrr*^*−/−*^/wild-type ranking of test-statistic values from a Cuffdiff2 differential analysis. Tiles are shaded based on the z-score of the test-statistic for genes within the given gene set, relative to all genes for a given condition to show direction of expression relative to wild-type. **DOI:** http://dx.doi.org/10.7554/eLife.01749.008

*Fendrr* is a 2,380 bp transcript consisting of six exons. It is transcribed from a bidirectional promoter shared with the protein coding gene *Foxf1a,* located 1,354 bp from its transcriptional start site (Figure 3A). An orthologous human *FENDRR (LINC–FOXF1)* transcript expressed from a syntenic region was identified within our catalog of human lincRNAs (Cabili et al., 2011). We previously demonstrated that this lincRNA is predominantly nuclear and physically associates with the PRC2 Polycomb complex (Khalil et al., 2009).

**Figure 3—figure supplement 1.**
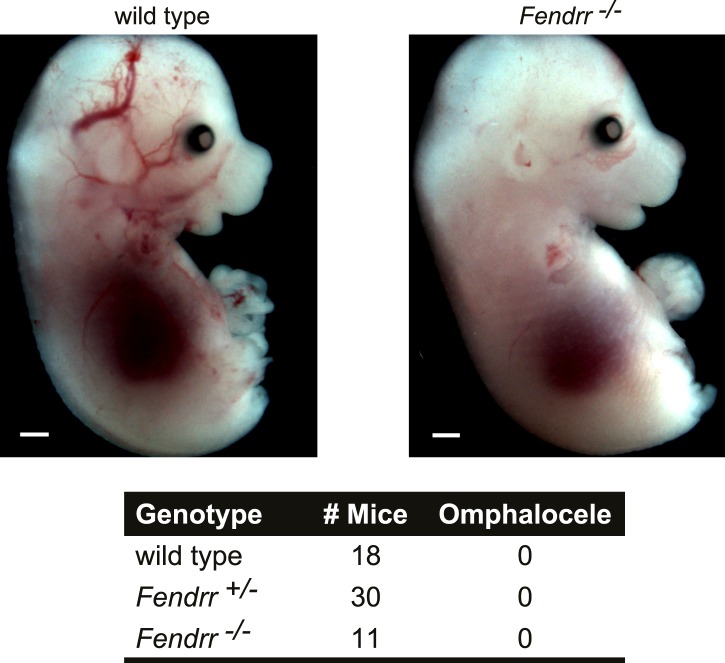
*Fendrr*^*−/−*^ embryos don’t have an omphalocele. Wild type and *Fendrr* mutant E14.5 embryos were harvested and examined for the presence of an omphalocele. Numbers of embryos analyzed for each genotype are indicated. Tail and limbs were removed. Scale bar = 1 mm. **DOI:** http://dx.doi.org/10.7554/eLife.01749.009

**Figure 3—figure supplement 2.**
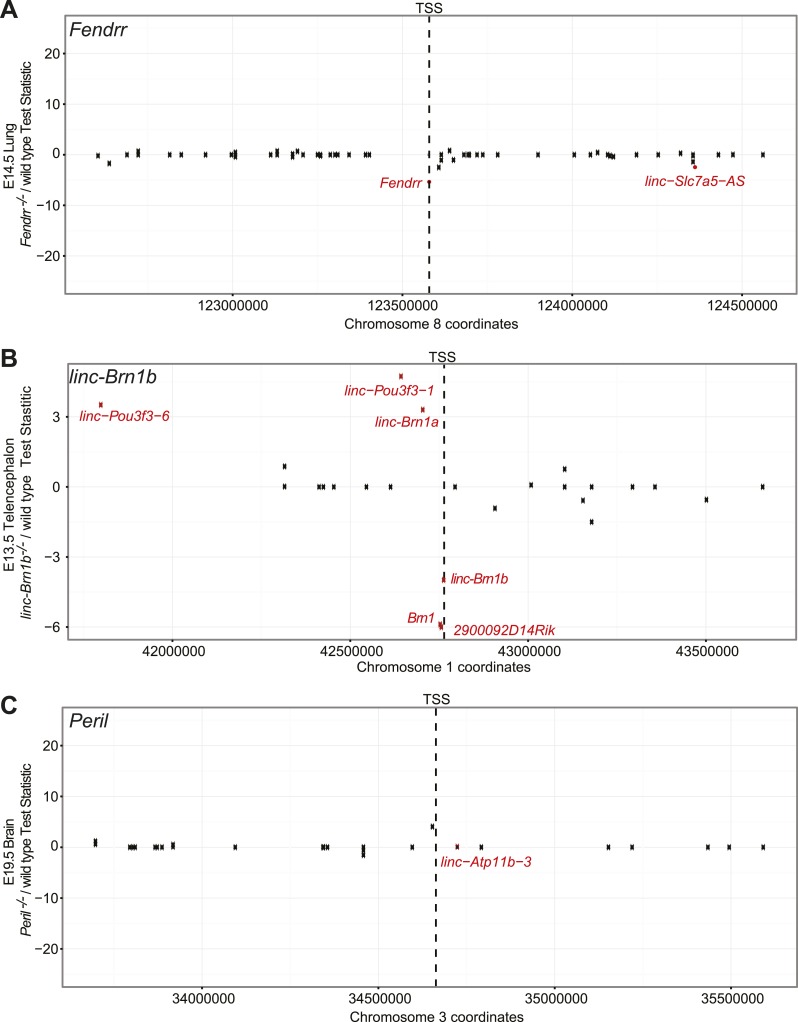
*Fendrr*, *linc–Brn1b*, and *Peril* do not act as cis-enhancer elements. Scatterplot showing Cuffdiff2 test-statistic (Knockout/wild type) for each gene ±1 Mb from the start site of the lincRNA. Genes with significant differential expression (q≤0.05) are highlighted in red. In each case, there is no significant enrichment for differentially expressed genes within the ±1 Mb window relative to the background distribution of differentially genes, as determined from random sampling of windows for each comparison. **DOI:** http://dx.doi.org/10.7554/eLife.01749.010

To determine the onset of *Fendrr* lethality, we monitored the survival of embryos at both early and late stages of embryonic development, as well as that of newborn pups. Normal Mendelian ratios were found at both E14.5 and E18.5 (Figure 3B), with embryos appearing macroscopically normal prior to birth (Figure 3C), suggesting that the lethality most likely occurred after birth. Importantly, we observed 8 *Fendrr*^*−/−*^ mutant newborns (P0) (Figure 3B), all of which died within 24 hr, indicating perinatal lethality.

During the course of our study, another group generated a *Fendrr* loss-of-function mouse model by replacing the first exon with multiple transcriptional termination sequences (Grote et al., 2013). In contrast to the results presented here, Grote et al. observed lethality at E13.75 due to heart and body wall (omphalocele) defects. When analyzing E14.5 embryos, we found no resorbed embryos or omphalocele in our *Fendrr* homozygous mutants (Figure 3—figure supplement 1). Although, both studies used similar genetic background strains, a possible explanation for this discrepancy may be found in the distinct targeting strategy used to remove the *Fendrr* gene. Regardless, both studies confirm that loss of *Fendrr* is lethal in mice.

Grote et al. observed *Fendrr* expression to be restricted to nascent lateral plate mesoderm and did not detect it in any other tissue. Using RNA-Seq expression profiling from adult mouse tissues and cell lines, we however found that *Fendrr* is expressed at high levels in the adult lung, and lower levels are detectable in colon, liver, spleen and brain (Figure 3D). Analysis of the knocked-in *lacZ* reporter in E14.5 and E18.5 embryos confirmed expression of *Fendrr* in these tissues as well as in the trachea and all along the gastrointestinal tract (Figure 3E,F). Interestingly, in the developing respiratory and digestive tracts at E14.5 and E18.5, expression of *Fendrr* is restricted to the pulmonary mesenchyme surrounding the bronchiolar epithelial cells (Figure 3E, right panels); in mesenchymal cells of the developing mucosa; the muscularis externa of the gut and in lymphoid aggregations within the gut’s mucosa (Figure 3F, bottom panel), all of which are derived from the lateral plate mesoderm.

Perinatal lethality is often associated with respiratory failure. Since the highest expression levels of *Fendrr* are found in the lungs, we evaluated initiation of breathing in surgically delivered E18.5 embryos. After cleaning of their airways, all *Fendrr*^*−/−*^ embryos analyzed either failed to breathe or gasped and stopped breathing within 5 hr (n = 7 *Fendrr*^−/−^, Figure 3G). In contrast, respiration initiated normally and was maintained for all but one of the heterozygote and wild-type embryos (n = 15 *Fendrr*^*+/−*^ and n = 8 wild type). *Fendrr*^*−/−*^ lungs at the pseudoglandular stage (E14.5) were hypoplastic compared to wild type (Figure 3H), and histological evaluation of the lungs revealed a decrease in the number and organization of pulmonary arteries, and a general failure of vasculogenesis within the lungs of the *Fendrr*^*−/−*^ mutants compared to wild type (n = 3 *Fendrr*^*−/−*^ and n = 3 wild type; Figure 3I, upper panels). At E18.5, *Fendrr*^*−/−*^ lungs appeared to have fewer but larger alveoli (n = 3 *Fendrr*^*−/−*^ and n = 3 wild type; Figure 3I, lower panels). Together, these results suggest that respiratory failure observed at birth in *Fendrr*^−/−^ mice could be due to a lung maturation and vascularization defect.

We also observed expression of *Fendrr* in the esophagus and gut. We observed thinning of the mesenchymal layer of the mucosa and external smooth muscle layers in the esophagus at E18.5 (n = 3 *Fendrr*^*−/−*^ and n = 3 wild type; Figure 3J). Although we did not observe *Fendrr* expression in the heart at E14.5, E18.5, or postnatally, we did observe intraventricular septal heart defects prior to birth (E18.5) (n = 3 *Fendrr*^*−/−*^ and n = 3 wild type; Figure 3K). In accordance with *Fendrr* having a previously described role in lateral plate mesoderm (Grote et al., 2013), our results suggest a more general role for *Fendrr* in regulating the proper differentiation of mesenchyme-derived tissues across several organ systems.

We next investigated if neighboring gene expression is perturbed by the deletion of *Fendrr*. We harvested lungs from E14.5 *Fendrr*^*−/−*^ embryos and wild-type littermates (n = 2) and performed differential RNA-Seq analyses. A loss of *Fendrr* expression in knockout relative to wild-type lungs confirmed deletion of *Fendrr* (Figure 3L). No significant change in the expression of the *Foxf1a* protein coding gene was observed in the *Fendrr*^−/−^ lung. Furthermore, genes within ±1 Mb of the *Fendrr* locus were not significantly differentially expressed any more than background levels of local enrichment (Figure 3—figure supplement 2A, p<0.087; bootstrapped from random 2 Mb genomic intervals), suggesting that the *Fendrr* gene does not act as a local enhancer. GSEA identified gene sets involved in muscle differentiation and contraction as the most significant sets misregulated in *Fendrr*^*−/−*^ lungs compared to wild type (Figure 3M). This agrees with our identification of defects in the lung vasculature of the *Fendrr*^*−/−*^ mice. Further studies will be needed to understand how specific changes in gene-expression patterns upon deletion of *Fendrr* contribute to the observed defects and perinatal lethality.

### Ablation of distinct lincRNA genes affects mouse growth

**Figure 4.**
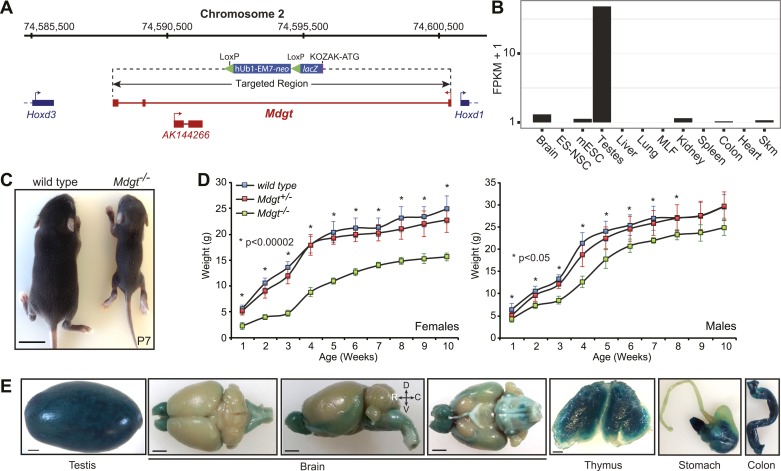
*Mdgt*^*−/−*^ surviving mice have growth defects. (**A**) *Mdgt* genomic locus and targeting scheme. (**B**) RNA-Seq expression profile for *Mdgt* across a panel of mouse tissues and cell types. (**C**) Representative example showing the reduced size of *Mdgt*^*−/−*^ pups compared to wild type 7 days after birth (P7). (**D**) Body weight (g) measurements over a 10 weeks period show growth retardation in both female and male *Mdgt*^*−/−*^ mice compared to wild type and *Mdgt*^*+/−*^ littermates (Females: n = 5 *Mdgt*^*−/−*^, n = 15 *Mdgt*^*+/−*^ and n = 10 wild types; Males: n = 3 *Mdgt*^*−/−*^, n = 10 *Mdgt*^*+/−*^ and n = 10 wild types). Significant p values at each time point are indicated (*). (**E**) Whole mount *lacZ* stainings reporting expression of *Mdgt* in adult tissues of heterozygote mutant mice. Scale bars = 1 mm, testis, thymus; 2 mm, brain, stomach, colon. **DOI:** http://dx.doi.org/10.7554/eLife.01749.011

To determine if deletion of our candidate lincRNAs affects normal development and growth postnatally, each strain was examined for gross morphological abnormalities and body weight (BW) measurements were taken. Homozygote mutant mice with heterozygote and wild-type littermate controls were compared over a 7–10 week period. *Mdgt*^*−/−*^ pups displayed a severe growth retardation phenotype, which may contribute to their lethality (Figure 4C,D). A week after birth, *Mdgt*^*−/−*^ pups were already significantly smaller than heterozygote and wild-type littermates (Figure 4C), with females 60% smaller (n = 5, p<0.00001) than wild-type littermates (n = 10) and males 32% smaller (n = 3, p<0.05), suggesting a sex bias in this phenotype. In *Mdgt*^*−/−*^ survivors, this retarded growth persisted up to 10 weeks in females, which were still 37% smaller than wild types (Figure 4D, p<0.0006). The defect, although milder, also persisted in male mutants up to at least 8 weeks.

**Figure 4—figure supplement 1.**
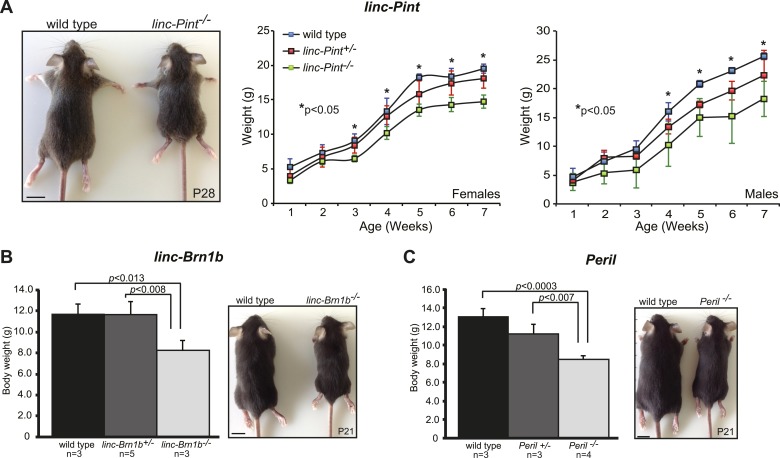
Growth retardation phenotype in lincRNA knockout strains. Body weight (g) measurements of wild type, lincRNA heterozygote and homozygote mutants for (**A**) *linc–Pint*, (**B**) *linc–Brn1b* and (**C**) *Peril* were taken at the indicated postnatal time points. Animals used for measurements (n) were derived from at least 2–3 litters. Paired Student's *t* test was used to assess statistical significance (p) in mean values. Scale bar = 1 cm. **DOI:** http://dx.doi.org/10.7554/eLife.01749.012

*Mdgt* is a 443 bp transcript consisting of three exons transcribed from a bidirectional promoter shared with the homeobox gene *Hoxd1*. The first exon for *Mdgt* is located only 84 bp away from the *Hoxd1* transcription start site. Another noncoding transcript, *AK144266,* is contained within an intron of *Mdgt* and appears to be expressed from the opposite strand (Figure 4A). In our targeting strategy, the entire *Mdgt* locus was deleted, including the *AK144266* transcript (Figure 4A). Expression estimates obtained from RNA-Seq revealed that *Mdgt* is highly expressed in testes, and more moderately in brain, kidney, colon and skeletal muscles (Figure 4B). *lacZ* staining confirmed expression in those tissues and also revealed expression in stomach and thymus (Figure 4E).

In addition to *Mdgt*, stunted growth defects were also observed in three other strains (Figure 4—figure supplement 1). 3 weeks after birth, *linc–Pint*^*−/−*^ mice were noticeably smaller than wild-type littermates (Figure 4—figure supplement 1A). By 7 weeks of age, females had 22% reduced body weight (n = 5, p<0.001) compared to wild type (n = 4), whereas males were 29% smaller (n = 3 *linc–Pint*^*−/−*^; n = 3 wild type, p<0.05). A decrease in body weight was also observed by three weeks of age in *linc–Brn1b*^*−/−*^ (29%, p<0.013, n = 3) and *Peril*^*−/−*^ mice (35%, p<0.0003, n = 4) when compared to wild-type littermates (n = 3) (Figure 4—figure supplement 1B,C). Together, these results indicate that specific lincRNAs can be important for normal body weight and growth.

### Identification of developmentally regulated human lincRNA orthologs

In order to determine whether loss of our candidate lincRNAs could lead to other developmental defects, we examined additional mutant strains that did not show perinatal lethality. Building on prior evidence that lincRNAs are abundantly expressed and spatio-temporally regulated within the brain during development and adulthood (Mercer et al., 2008; Qureshi et al., 2010; Cabili et al., 2011; Ramos et al., 2013), we concentrated on the candidate lincRNAs that were expressed in the brain or in neural progenitors. Twelve of the lincRNAs for which we have established knockout strains exhibited such expression profile (Figure 1B). To focus on lincRNA candidates of potential functional relevance in neuronal development, we used syntenic orthology (TransMap [Zhu et al., 2007]) and RNA-sequencing to select those with identifiable human orthologs, and whose expression was regulated during in vitro neural differentiation.

**Figure 5.**
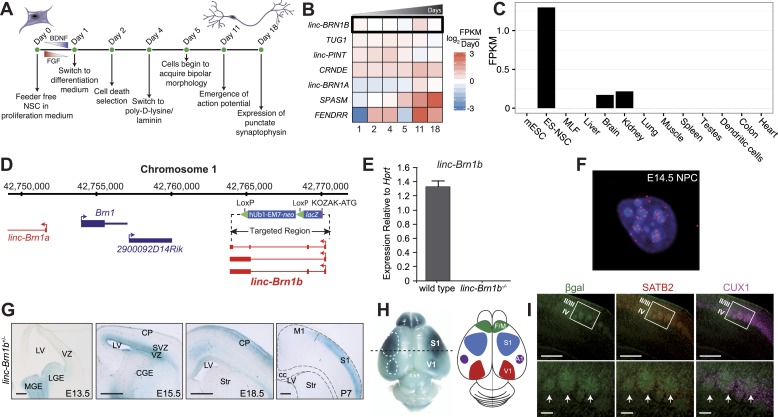
*linc–Brn1b* is spatio-temporally regulated during corticogenesis. (**A**) Schematic overview of the in vitro human neural stem cell differentiation protocol. RNA was collected at the indicated time points and sequenced to identify significantly differentially expressed lincRNA human orthologs. (**B**) Heatmap of log_2_ ratios to undifferentiated (Day 0) human neural stem cells for 7 of the 20 lincRNAs selected for deletion with a significant (q<0.01; Cuffdiff2) increase in expression during differentiation. (**C**) RNA-Seq expression profile for *linc–Brn1b* across a panel of mouse tissues and cell types. (**D**) *linc–Brn1b* genomic locus and targeting strategy. (**E**) qRT–PCR confirmation of the genotype for both heterozygotes (+/−) and homozygous null (−/−) mutants. (**F**) Single-molecule RNA FISH targeting *linc–Brn1b* in wild-type E14.5 cortical neurospheres. (**G**) *lacZ* staining shows expression profile of *linc–Brn1b* at different embryonic (E13.5, E15.5 and E18.5) and early postnatal stages (P7) in *linc–Brn1b*^*+/−*^ telencephalon. *lacZ* expression in neural progenitors of both ventral telencephalon (lateral ganglionic eminence, LGE and medial ganglionic eminence, MGE) (E13.5) and dorsal telencephalon (ventricular zone, VZ and subventricular zone, SVZ) (E15.5) is detected. Restricted expression in the upper cortical layers is observed in both E18.5 and P7 cortex. (**H**) Whole mount *lacZ* staining of P7 *linc–Brn1b*^*+/−*^ brain shows distinct *linc–Brn1b* expression in primary somatosensory (S1) and visual (V1) cortical areas. (**I**) β-galactosidase immunofluorescence on coronal sections of P7 *linc–Brn1b*^*−/−*^ cortex shows *linc–Brn1b* expression in layer IV of the somatosensory area (white boxes), specifically within the barrel structures (white arrows), as determined by co-staining with the upper layer markers SATB2 and CUX1. Abbreviations: LV, lateral ventricle; LGE, lateral ganglionic eminence; MGE, medial ganglionic eminence; CGE, caudal ganglionic eminence; CP, cortical plate; SVZ, subventricular zone; VZ, ventricular zone; Str, striatum; M1, primary motor area; S1, primary somatosensory area; cc, corpus callosum; V1, primary visual cortex; A1, primary auditory cortex; F/M, frontal motor cortex. Scale bars: 500 µm (**G**), (**I**, upper panels); 100 µm (**I**, lower panels). **DOI:** http://dx.doi.org/10.7554/eLife.01749.013

Briefly, transcripts expressed along a time course of ES-derived human neural stem cells (NSC) differentiation (Goff et al., 2009) (Figure 5A) were assembled and aggregated with an existing compendium of RNA-Seq data, using our previously described lincRNA discovery pipeline (Cabili et al., 2011). The resulting lincRNA catalog contained 24,737 distinct, high-quality transcript reconstructions corresponding to 14,259 human lincRNA genes. We observed 769 lincRNA genes with significant differential expression (q<0.01; Cuffdiff2) between any two adjacent time points during NSC differentiation. With a false discovery rate of 1% (Figure 5—figure supplement 1), 302 of these were significantly induced relative to day 0 (Figure 5—figure supplement 1). This approach revealed that 7 lincRNAs from our mouse knockout strains have human orthologs that are dynamically induced during in vitro human neuronal differentiation (Figure 5B). Interestingly, two of these, *linc–Brn1a* and *linc–Brn1b*, were almost exclusively expressed in NSCs as determined by RNA-Seq (Figure 1B). These lincRNAs reside in the genomic region of *Brn1* (*Pou3f3*), a well-studied transcription factor involved in cortical development (McEvilly, 2002; Sugitani et al., 2002; Dominguez et al., 2012). We thus focused on this locus, starting with *linc–Brn1b,* as it is not transcribed from a bidirectional promoter and therefore deletion does not disrupt the *Brn1* promoter.

**Figure 5—figure supplement 1.**
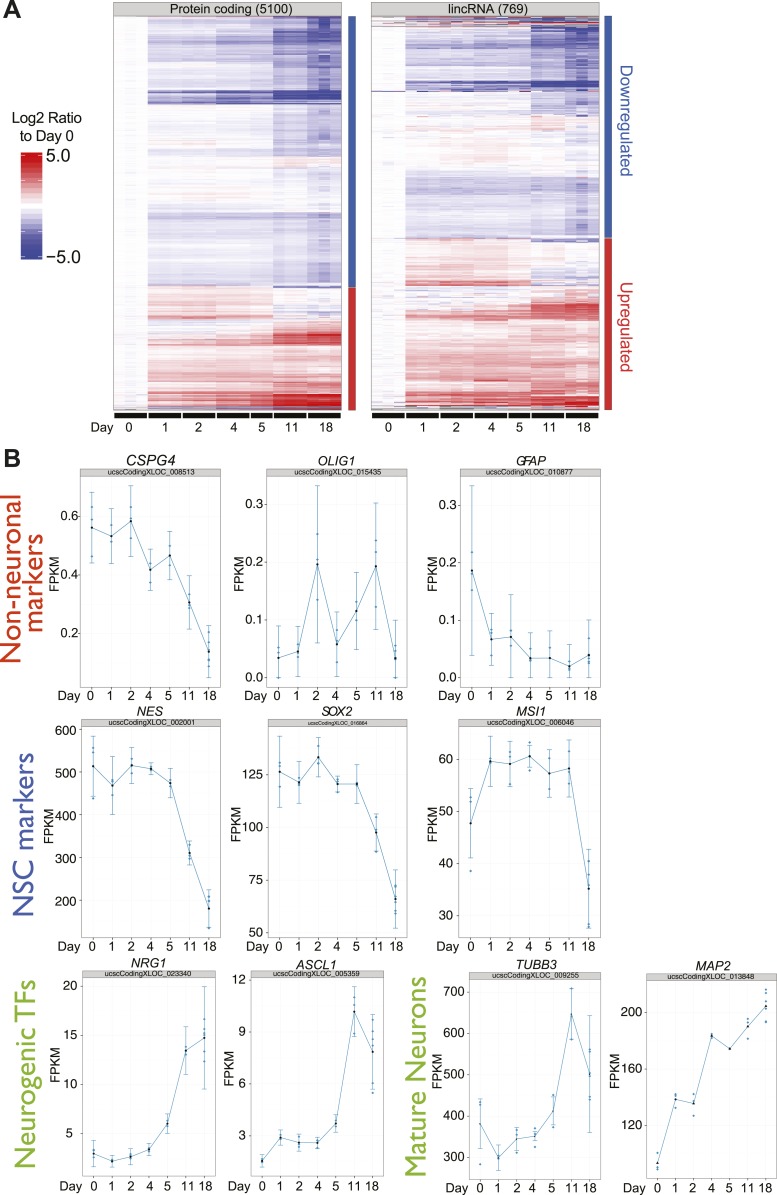
Differential expression of protein coding genes, lncRNAs and neuronal markers during human ES-derived neuronal differentiation time course. (**A**) Heatmap of Cuffdiff2 estimated expression values (FPKM) expressed as log2 fold-change to Day 0 for 5,100 significant protein-coding genes and 769 significant lncRNAs (q<0.001; Cuffdiff2) from a human H1-derived NSC differentiation timecourse. (**B**) Individual expression plots for key neural stem cell markers, neuronal markers, and non-neuronal markers confirm the differentiated state of the human H1-derived NSC into predominantly neurons. **DOI:** http://dx.doi.org/10.7554/eLife.01749.014

### *Linc–Brn1b* expression is spatio-temporally restricted in the developing cerebral cortex

Using RNA-Seq data, we observed *linc–Brn1b* to be predominantly expressed in ES-derived NSCs with additional moderate expression in the adult brain and kidney (Figure 5C). The *linc–Brn1b* gene is a ∼3 Kb transcript derived from a 6.8 Kb genomic locus approximately 10 Kb downstream of the *Brn1* protein coding gene. To generate the *linc–Brn1b* knockout mice, we targeted the entire *linc–Brn1b* locus (Figure 5D). Complete ablation was confirmed by qRT–PCR (Figure 5E) using adult brain cDNA as template. RNA-FISH in mouse E14.5 neural progenitor cells (NPCs) isolated from the cerebral cortex demonstrated that the *linc–Brn1b* transcript is predominantly nuclear with moderate cytoplasmic expression (Figure 5F).

While identified as a lincRNA expressed in adult brain, the spatio-temporal distribution of *linc–Brn1b* during brain development is not known. Therefore, we used *lacZ* expression from the *linc–Brn1b* locus in heterozygote mutants to define its expression in vivo. We found that *linc–Brn1b* was expressed within neural progenitors of both the ventral and dorsal telencephalon, as early as E13.5 (Figure 5G). Detailed characterization of expression in the germinal zones of the dorsal telencephalon showed that by E15.5, it was strongly expressed in progenitors of both the ventricular zone (VZ) and the subventricular zone (SVZ) of the developing cerebral cortex, and by E18.5 showed restricted expression in the developing upper cortical layers. In addition, whole-mount *lacZ* staining at P7, showed a specific areal distribution for *linc–Brn1b* within both the primary somatosensory cortex and the primary visual cortex (Figure 5H). Within the somatosensory cortex, *linc–Brn1b* was expressed in the barrel structures of the posteromedial barrel subfield (PMBSF) (Figure 5I); a highly organized region of cortical projection neurons that receives afferent connections from the thalamus and is responsible for the coordination of sensory inputs from the rodent vibrissae (Petersen, 2007).

Collectively, the timing and location of *linc–Brn1b* expression within the developing cortex suggests a potential role for *linc–Brn1b* in area-specific development of distinct classes of projection neurons.

### Loss of *linc–Brn1b* results in specific reduction in the number of intermediate progenitor cells (IPCs) in the cerebral cortex

**Figure 6.**
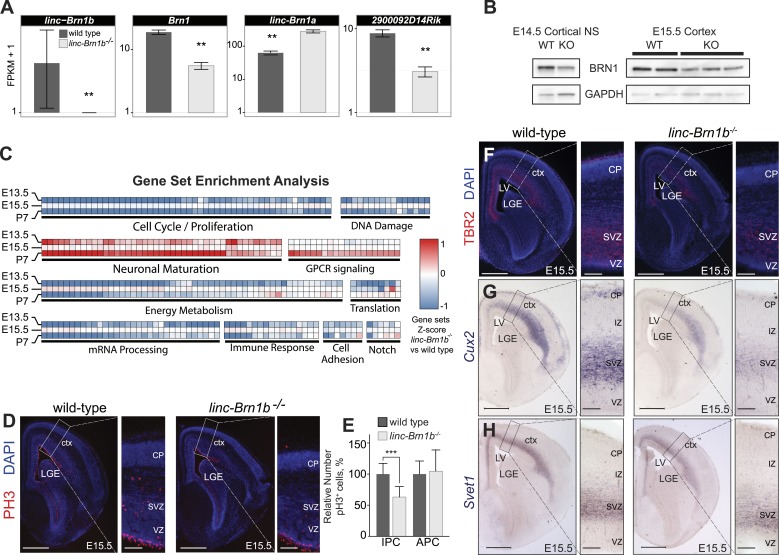
*linc–Brn1b*^*−/−*^ mice demonstrate defects in proliferation of IPCs. (**A**) RNA-Seq expression estimates from E15.5 wild type and *linc–Brn1b*^*−/−*^ telencephalon for *linc–Brn1b*, the protein coding gene *Brn1*, and neighboring genes *linc–Brn1a* and *2900092D14Rik*. (**B**) Western blots of wild type and *linc–Brn1b*^*−/−*^ E14.5 cortical neurospheres (NS), and E15.5 cortices. (**C**) GSEA of *linc–Brn1b*^*−/−*^ vs wild type in E13.5 and E15.5 telencephalon and P7 whole brain (as described in Figure 3). (**D** and **E**) Immunofluorescence staining for the mitotic marker phosphorylated histone H3 (pH3) in E15.5 coronal sections of cortex (**D**) and pH3^+^ cell counts of apical progenitors (APC) and intermediate progenitors (IPC) (**E**). (**F**–**H**) Immunofluorescence staining for TBR2 (**F**), and in situ hybridization for *Cux2* (**G**) and *Svet1* (**H**) in wild type and *linc–Brn1b*^*−/−*^ E15.5 cortex show that mutant mice have decreased expression of SVZ intermediate progenitor markers. Scale bars = 500 µm (**D**–**H**). **p<0.01, ***p<0.001; Student’s *t* test. LGE, lateral ganglionic eminence; LV, lateral ventricule; ctx, cortex; CP, cortical plate; VZ, ventricular zone; SVZ, subventricular zone; IZ, intermediate zone. **DOI:** http://dx.doi.org/10.7554/eLife.01749.015

To investigate the consequences of genetic deletion of *linc–Brn1b* during development of the telencephalon, we performed RNA-Seq on E13.5 and E15.5 whole telencephalons and P7 whole brains (n = 2) of wild type and *linc–Brn1b*^*−/−*^ mice. At all time points, RNA-Seq analyses identified a statistically significant reduction (∼50%) in expression of the neighboring *Brn1* protein coding gene in *linc–Brn1b*^*−/−*^ brains relative to wild type (E15.5 shown in Figure 6A, Figure 6—figure supplement 1A,B). We observed a similar decrease in BRN1 protein expression between wild type and *linc–Brn1b*^*−/−*^ E14.5 cortical-derived neurospheres and E15.5 whole cortex samples (Figure 6B). In contrast, upon knockout of *linc–Brn1b,* we observed a significant increase in expression of *linc–Brn1a* (p<0.01; Cuffdiff2), which shares a bidirectional promoter with *Brn1* (Figure 6A). This suggests opposing regulatory effects on the neighboring lincRNA and protein coding genes upon deletion of *linc–Brn1b.*

**Figure 6—figure supplement 1.**
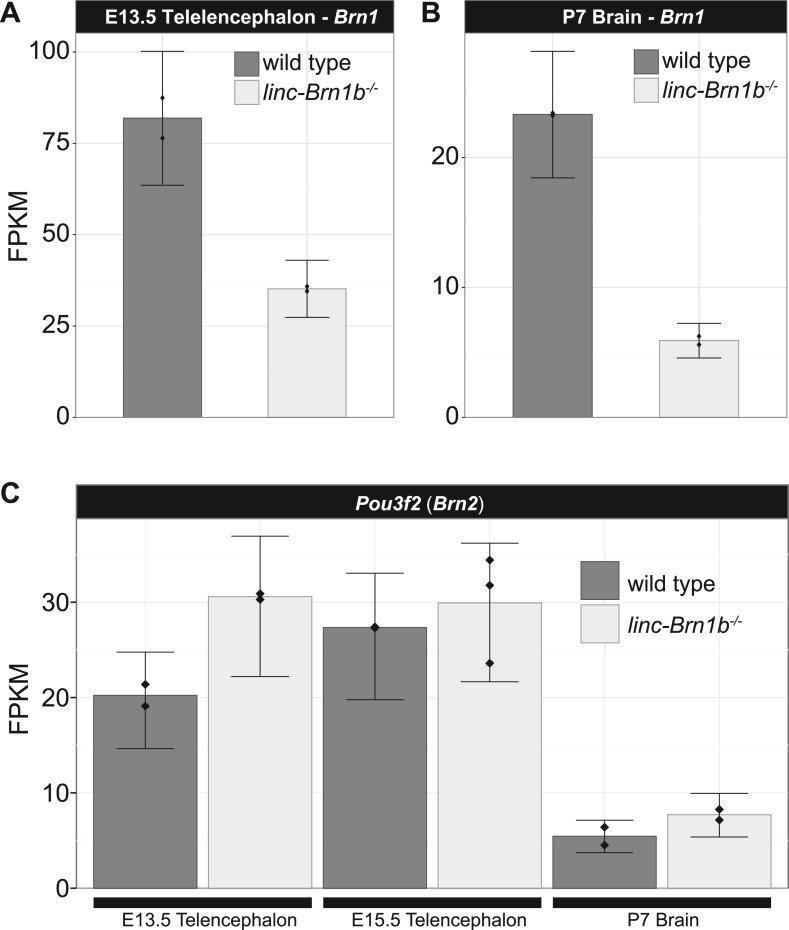
*Brn1* transcript is reduced in *linc–Brn1b*^*−/−*^ E13.5 telencephalon and P7 brain. RNA-Seq expression estimates (FPKM) for the *Brn1* protein coding gene in E13.5 telencephalon (**A**) and P7 brain (**B**) harvested from wild type and *linc-Brn1b^-/-^* littermates (n=2). (**C**) The *Brn1* paralog *Brn2* has no significant changes in expression in the *linc-Brn1b^-/-^* mice in any condition, suggesting no contribution from this gene to the observed phenotype. **DOI:** http://dx.doi.org/10.7554/eLife.01749.016

Gene set enrichment analysis (GSEA) revealed a significant decrease in genes associated with cellular growth and proliferation in *linc–Brn1b*^*−/−*^ relative to wild type at all three time points sampled and an increase in gene sets positively correlated with neuronal maturation (Figure 6C). These results are consistent with a predicted role for *linc–Brn1b* derived from our initial GBA analyses (Figure 1C) and suggest a function for *linc–Brn1b* in the differentiation of neural progenitors and neurogenesis in the telencephalon.

To investigate potential abnormalities of *linc–Brn1b*^*−/−*^ mutants, we concentrated on the dorsal telencephalon (the developing cerebral cortex). First, to quantify the proliferation of cortical progenitors, we evaluated the expression of the mitotic marker phosphorylated histone H3 (pH3) in E15.5 wild type and *linc–Brn1b*^*−/−*^ cortices (Figure 6D). Quantification of pH3^+^ cells revealed a significant and specific reduction in the relative percentage of pH3^+^ intermediate progenitor cells within the SVZ (36.88% decrease; p<3.93e-05; Student’s *t* test) in *linc–Brn1b* mutant vs control cortices (Figure 6E). The observed abnormalities were specific to intermediate progenitors, as there was no statistically significant change in the proliferation rate of apical progenitors within the VZ (Figure 6E).

In agreement with a reduction in intermediate progenitors of the cortical SVZ we found decreased expression of the markers TBR2 (Figure 6F), *Cux2* (Figure 6G), and *Svet1* (Figure 6H) (Tarabykin et al., 2001; Zimmer, 2004; Molyneaux et al., 2007; Arnold et al., 2008; Cubelos et al., 2008; Sessa et al., 2008) in *linc–Brn1b*^*−/−*^ mutants.

Together, the data indicate that *linc–Brn1b* knockout mice exhibit a decrease in proliferation of cortical progenitors in vivo, and that this defect is restricted to a specific subpopulation of progenitors in the SVZ of the developing cortex.

### Cortical lamination is abnormal in *linc–Brn1b^−/−^* mice

To investigate whether the observed decrease in SVZ progenitors resulted in an overall reduction in cortical thickness, we measured *linc–Brn1b*^*−/−*^ mutants and wild-type littermates at P7. The distance between the pia and the white matter was measured at matched medio-lateral and rostro-caudal locations (n = 17 wild-type sections and n = 10 *linc–Brn1b*^*−/−*^ sections). We reproducibly observed a significant 6.24% decrease in the total thickness of the neocortex in the mutants, relative to wild type (1317.31 µm ± 20.03 in *linc–Brn1b*^*−/−*^ vs 1404.90 µm ± 15.07 in wild type; p<0.002, Student’s *t* test) (data not shown)*.*

**Figure 7.**
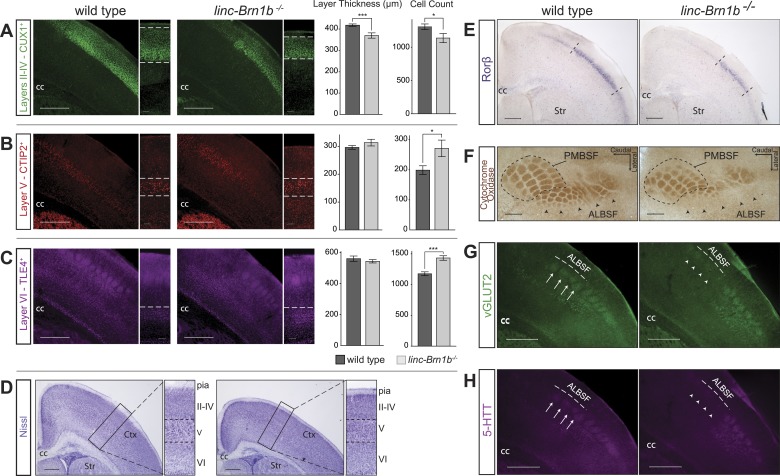
Abnormal cortical lamination and disruption of the barrel cortex in *linc–Brn1b*^*−/−*^ mice. (**A**) Immunofluoresence staining and quantification on coronal sections of P7 wild type and *linc–Brn1b*^*−/−*^ cortices for upper layer II–IV marker CUX1 show a significant reduction in absolute layer thickness and total number of CUX1^+^ projection neurons in *linc–Brn1b*^*−/−*^ mice. (**B** and **C**) Immunofluorescence staining and quantification on coronal sections of P7 wild type and *linc–Brn1b*^*−/−*^ cortices for layer V marker CTIP2 (**B**), and for layer VI marker TLE4 (**C**) show not significant change in the total thickness of layer V and VI, but significant increase in the number of CTIP2^+^ subcerebral projection neurons and TLE4^+^ corticothalamic neurons in *linc–Brn1b*^*−/−*^ mice. (**D**) Nissl staining of coronal sections of P8 wild type and *linc–Brn1b*^*−/−*^ cortices shows overall cortical reduction and specific decrease in upper layer thickness in *linc–Brn1b*^*−/−*^ mice. (**E**) *Rorβ* in situ hybridization on coronal sections of P7 wild type and *linc–Brn1b*^*−/−*^ primary somatosensory cortex. (**F**) Cytochrome oxidase c activity on sections spanning barrel cortex shows reduction in the anteriolateral barrel subfield (ALBSF) in *linc–Brn1b*^*−/−*^ mice. The loss of barrels in the ALBSF and their general disorganization are confirmed by immunofluorescence staining for vGLUT2 (**G**) and 5-HTT (**H**). The full arrows indicate individual barrels and arrowheads point to corresponding barrels that are absent in the *linc–Brn1b*^*−/−*^ brains. *p<0.05, **p<0.01, ***p<0.001; Student’s *t* test. Scale bars = 500 µm (**A**–**E**) and (**G**–**H**), 100 µm (**F**). ctx, cortex; cc, corpus callosum; str, striatum; PMBSF and ALBSF, posteriomedial and anteriolateral barrel subfield. **DOI:** http://dx.doi.org/10.7554/eLife.01749.017

To define whether generation of projection neuron subtypes was affected in the absence of *linc–Brn1b*, cortices from *linc–Brn1b*^*−/−*^ and wild-type littermates were collected at P7 and immunostained for markers of different projection neuron classes: CUX1, a marker of upper layer II/III and IV callosal projection neurons (Zimmer, 2004) (Figure 7A), CTIP2, a marker of layer V subcerebral projection neurons (Arlotta et al., 2005) (Figure 7B), and TLE4, a marker of layer VI corticothalamic projection neurons (Koop et al., 1996; Chen, 2005; Molyneaux et al., 2005) (Figure 7C). Measurements of the thickness of each cortical layer showed a distinct reduction in the upper layer II/III–IV (11.62% reduction in absolute thickness in *linc–Brn1b*^*−/−*^ vs wild type; Figure 7A, p<0.0004; Student’s *t* test), which was also detectable by histological Nissl staining (Figure 7D). In contrast, no significant change in the absolute thickness of either layer V (Figure 7B, p<0.18; Student’s *t* test) or VI (Figure 7C, p<0.54; Student’s *t* test) was detected.

In order to determine whether the number of specific classes of neurons within layers was affected in the mutant cortex, we quantified the percentage of CUX1^+^, CTIP2^+^ and TLE4^+^ neurons. In agreement with a reduction in the thickness of layer II/III–IV, we observed that the number of CUX1^+^ callosal projection neurons was decreased by 12.97% in the mutant compared to wild type (Figure 7A, mean = 1137 ± 69 CUX1^+^ cells in *linc–Brn1b*^*−/−*^ vs mean = 1307 ± 52 in wild type; p<0.03; Student’s *t* test). Conversely, despite the unchanged thickness of layers V and VI, the number of CTIP2^+^ subcerebral projection neurons (Figure 7B, mean = 271 ± 28 in *linc–Brn1b*^*−/−*^ vs mean = 199 ± 14 in wild type; p<0.016; Student’s *t* test) and TLE4^+^ corticothalamic projection neurons (Figure 7C, mean = 1432 ± 37 in *linc–Brn1b*^*−/−*^ vs mean = 1176 ± 31 in wild type; p<5.9 × 10^−5^; Student’s *t* test) neurons were increased by 36.39% and 21.77% in the *linc–Brn1b*^*−/−*^ mice, respectively. These data suggest the possibility that a subset of upper layer progenitors abnormally generate deep layer neurons in place of CUX1^+^ upper layer neurons in the *linc–Brn1b*^*−/−*^ mutant.

The selective reduction in the generation of upper layer cortical neurons is interesting, considering that these neurons are principally derived from the expansion of intermediate progenitors, which are reduced in *linc–Brn1b*^*−/−*^ mice. Taken together, these results indicate that *linc–Brn1b* is required for proper generation of cortical neurons, in particular projection neurons of layer II/III–IV.

### Disorganization of the barrel cortex in *linc–Brn1b*^*−/−*^ mice

Whole mount analysis of *linc−Brn1b* areal distribution showed high levels of expression in primary somatosensory cortex, an area that in rodents receives thalamocortical afferents relaying sensory information from the mustacial vibrissae (Petersen, 2007). Given the expression of *linc–Brn1b* within this region, and the requirement for proper specification of upper layer neurons, we investigated whether *linc–Brn1b* is required for the proper development of the somatosensory cortex and organization of the barrel structures. In situ hybridization against the barrel cortex marker RAR-related orphin beta (*Rorβ*) (Jabaudon et al., 2012), on matched coronal sections of P7 wild type and knockout mouse cortices demonstrated a reduction in the total size of the barrel cortex in the *linc–Brn1b*^*−/−*^ mice (Figure 7E) with a more pronounced loss of *Rorβ*^*+*^ neurons at the medial edge.

Histochemical staining of cytochrome oxidase C activity in the somatosensory cortex was examined and showed a distinct disruption of the individual barrel structures, particularly within the anteriolateral barrel subfield (ALBSF) (Figure 7F). A reduction in overall area and number of barrels in *linc–Brn1b* mutants was also observed in the highly organized posteriomedial barrel subfield (PMBSF). These findings, already consistent with the *Rorß* in situ hybridization data, were confirmed in P7 coronal sections immunostained with vGLUT2 (Figure 7G) and 5-HTT (Figure 7H), both of which specifically label the barrel structures. Analysis of both of these markers corroborates a specific impairment of the barrels within the ALBSF, and a general disorganization of individual barrel structures in the *linc–Brn1b*^*−/−*^ mice.

Taken together, these results demonstrate the requirement of *linc−Brn1b* for the proper development of different classes of projection neurons within the cerebral cortex, and suggest that the loss of *linc−Brn1b* may potentially have broader implications for state-dependent cortical sensory processing.

## Discussion

In the post genomic era, thousands of long noncoding RNAs have been discovered as transcribed units in mammalian genomes. However, what fraction of these new transcripts have general functional significance in vivo is debated. While several studies have indicated a role for lincRNAs in diverse biological processes (Ponting et al., 2009; Rinn and Chang, 2012; Mercer and Mattick, 2013; Ulitsky and Bartel, 2013), it has been suggested that most transcripts could represent nonfunctional transcriptional by-products (Struhl, 2007; Kowalczyk et al., 2012). Early critical studies of knockout strains (e.g., *Xist* and *Tsix*) did find lncRNAs implicated in X inactivation to be required for life. Yet, of the relatively few lncRNA mouse models derived since, many have displayed subtle defects or no phenotype (Ripoche et al., 1997; Gordon et al., 2010; Anguera et al., 2011; Nakagawa et al., 2011; Zhang et al., 2012).

Other strategies using RNAi and xenografts to assess the function of lncRNAs in vivo have revealed interesting roles in development and tumor growth (Ulitsky et al., 2011; Wang et al., 2011; Yang et al., 2013). Together with difficulties in finding a phenotype in mouse models such as *Malat1*, *Neat1* (Nakagawa et al., 2011; Zhang et al., 2012), these findings have led some to suspect that acute inactivation of lncRNAs leads to stronger phenotypes than constitutive deletions, where compensatory events may occur. Therefore, our study, grounded in genetic deletions, demonstrates the important physiological insights that can be gleaned by constitutive lincRNA knockouts.

By leveraging our large-scale RNA-sequencing and genomics studies in combination with an integrated and multifaceted candidate selection pipeline, we were successful in finding physiologically essential lincRNAs. Through the initial characterization of the 18 lincRNAs knockout strains generated here, we found three that exhibit peri- or post-natal lethality and two additional ones with distinct developmental defects. Future studies will describe in detail other promising phenotypes.

A few recent studies have demonstrated that lincRNAs regulate neighboring protein coding genes and thereby may function as *cis*-enhancers or *cis*-regulatory elements (Ørom et al., 2010; Wang et al., 2011). Here, we were able to examine several lincRNAs and the regulation of the neighboring protein coding genes in a genetically defined context. We find one instance where the deletion of a lincRNA, *Fendrr*, phenocopies the neighboring protein coding gene despite a non-significant effect on *Foxf1* gene expression. Interestingly, chromatin predictions of enhancers (Shen et al., 2012) suggest this lincRNA may have enhancer-like properties (H3K4me1 and Pol II). However, our genetic analysis is not consistent with the notion of *Fendrr* as a chromatin signature-defined ‘enhancer’. Specifically, Grote et al observed a similar lethality phenotype in *Fendrr* mutants, yet their strategy (insertion of a strong transcription stop sequence) retained almost the entire DNA segment of *Fendrr*. This suggests that *Fendrr* cannot be acting simply as a DNA enhancer, although we cannot rule out the passive act of transcription as being functional. In our model, both the endogenous promoter and first exon remain. Moreover, the introduced *lacZ* reporter is actively transcribed as well, thus ruling out the passive act of transcription for *Fendrr* activity. Finally, since this lincRNA does not appear to act as a local enhancer and does not encode a small peptide based on PhyloCSF and Ribosome profiling analyses, we believe *Fendrr* to be a physiologically relevant and functional RNA molecule.

Interestingly, *Fendrr* mutant mice exhibit many features of *Foxf1a* protein coding gene heterozygous mutants (Mahlapuu et al., 2001). Since we do not observe a significant decrease in *Foxf1a* expression in *Fendrr* mutants, this raises the possibility that *Fendrr* could act downstream of *Foxf1a*. The FOXF1A protein plays an important role in the development of the lung and the gastrointestinal tract (Mahlapuu et al., 2001). Similarly, we found that *Fendrr* is expressed in the mesenchyme of these tissues and that homozygote mutant mice display lung and gastrointestinal tract defects most likely leading to perinatal lethality. Several cases of genetic deletions encompassing the neighboring protein coding gene *FOXF1*, and also *FENDRR* have been observed in neonates with the lethal lung disorder ‘alveolar capillary dysplasia with misalignment of pulmonary veins’ (ACD/MPV) (Szafranski et al., 2013). This supports our hypothesis that lincRNAs can be required for normal organ development and misregulated in pathological states, which could translate to human disease.

Deletion of both *Peril* and *Mdgt* also results in viability defects. *Mdgt* is transcribed from a bidirectional promoter shared with *Hoxd1*, which is located only 84 bp away from *Mdgt*. Although we do not exclude that *Hoxd1* expression could be affected by our *Mdgt* deletion strategy, the phenotype of homozygous *Hoxd1* mutants, which are viable and fertile, is in sharp contrast with *Mdgt*^*−/−*^ mutants (Guo et al., 2010). Thus, deletion of *Mdgt* does not phenocopy *Hoxd1*, indicating a distinct function for this lincRNA. Interestingly, mice with a deletion of the adjacent *Hoxd3* protein coding gene display reduced viability similar to *Mdgt* (Condie and Capecchi, 1993). However, contrary to *Mdgt*^*−/−*^, the surviving *Hoxd3*^*−/−*^ mice do not appear to have a growth defect, again suggesting a distinct function for *Mdgt*.

Neither the *Peril* nor *Mdgt* locus appears to be enriched for the signature enhancer modifications H3K4me1, H3K27ac in any public mouse ENCODE datasets. In the case of *Peril*, no genes were found significantly differentially expressed in knockout vs wild-type brains within ±1 Mb of *Peril* (Figure 3—figure supplement 2C; p<1.0, bootstrapped from 1000 random genomic intervals). Therefore, cis effects, as suggested by other enhancer-associated lincRNAs like *Hottip* (Wang et al., 2011), are unlikely. Collectively, these results suggest that lethality of the three lincRNAs observed in this study are likely not due to *cis* or enhancer-like RNA regulatory effects.

In addition to viability phenotypes, we also describe several developmental abnormalities such as body size and cortical defects in the *linc–Brn1b* mutant strain. Based on currently available public data, the *linc–Brn1b* locus does not appear to have enrichment for chromatin marks characteristic of enhancers, but rather the canonical H3K4me3/H3K36me3 signature of active transcription in the brain. In this case, however, deletion of *linc–Brn1b* did affect the expression of the neighboring protein coding gene. *linc–Brn1b* resides near *Brn1* (*Pou3f3*), a key transcription factor that shares redundant roles with the closely related paralog *Brn2* (*Pou3f2*) in upper layer cortical development. Interestingly, deletion of *linc–Brn1b* results in a significant reduction in the neighboring BRN1 protein (Figure 6). However, in contrast to *linc–Brn1b* mutants, deletion of *Brn1* alone does not lead to defects in cortical lamination. Only when both *Brn1* and *Brn2* are deleted (*Brn1/2* double mutants) is a reduction in layers II–IV neurons observed (McEvilly, 2002; Sugitani et al., 2002; Dominguez et al., 2012). Despite the differential regulation of the adjacent *Brn1* gene, *linc–Brn1b* does not appear to act as a general *cis*-enhancer. The region has five other genes within ±1 Mb that are differentially expressed. However, this is not significant given the dramatic changes to the transcriptome in the *linc–Brn1b*^−/−^ E13.5 telencephalon (Figure 3—figure supplement 2B; p<0.225, bootstrapped from 1000 random genomic intervals).

We do not observe a significant difference in expression of the paralogous *Brn2* protein coding gene in any *linc–Brn1b*^−/−^ developmental stage analyzed when compared to wild type (Figure 6—figure supplement 1C), Thus, despite an observed decrease in *Brn1* expression in *linc–Brn1b*^*−/−*^*,* deletion of *linc–Brn1b* does not phenocopy deletion of the neighboring protein coding gene *Brn1*. In fact, ablation of *linc–Brn1b* results in a stronger phenotype than that observed for the neighboring protein coding gene, suggesting additional roles for this lincRNA. In addition, we observe expression of *linc–Brn1b* in regions of the brain with no known expression of *Brn1*. Together, these observations suggest that lincRNAs adjacent to important developmental regulators may act on upstream and/or distinct pathways in addition to already reported *cis*-enhancer-like mechanisms (Ørom et al., 2010; Wang et al., 2011).

Further leveraging our genetically defined deletion we gleaned additional insights into lincRNA biology and transcriptional regulation in vivo. It has been noted that lincRNAs have a propensity to be transcribed from bidirectional promoters (Cabili et al., 2011; Pauli et al., 2012; Ulitsky and Bartel, 2013). New studies have also suggested that many lincRNAs transcribed from bidirectional promoters are unstable and likely non-functional transcripts (Almada et al., 2013; Sigova et al., 2013). Interestingly, *Fendrr* is transcribed from a bidirectional promoter. Here, we observe that deletion of *Fendrr* is lethal despite leaving an intact promoter, expression of the first exon and not interfering with Pol II dynamics at this locus. Similarly, RNA-Seq analysis of *linc–Brn1b* knockout revealed a strong and significant upregulation of *linc–Brn1a,* a lincRNA transcribed from a bidirectional promoter shared with *Brn1*. This incongruous activity on adjacent genes (decrease in *Brn1* protein coding gene and increase in *linc–Brn1a* transcribed from a bidirectional promoter with *Brn1*) observed in the *linc–Brn1b*^*−/−*^ brain is suggestive of a functional role for the dynamically regulated *linc–Brn1a*, rather than transcriptional noise from bidirectional transcription from the protein coding gene promoter. Together, these findings confirm that not all lincRNAs transcribed from a bidirectional promoter are irrelevant transcriptional by-products, but rather suggests that some fraction of these transcripts play critical functional roles during development.

Our framework for lincRNA candidate selection for genetic analysis, based on RNA-sequencing catalogs and genomic studies, has led us to unexplored roles for lincRNAs in brain development. The brain (and the CNS more broadly) constitutes one of the most complex and fast evolving organs in the body. Here, it is likely that complex regulatory mechanisms of lincRNAs have evolved to build the layered control of gene expression necessary to generate the unparalleled cellular diversity and complex function of this organ. *linc–Brn1b* represents an example of a lincRNA that has an effect on development of the cerebral cortex. Beyond *linc–Brn1b*, many more lincRNAs from our screen have similar restricted patterns within progenitors in the VZ and SVZ of the telencephalon, possibly suggesting roles in neurogenesis. Others have highly cell-specific and dynamic in vivo expression patterns in distinct regions of the brain (Mercer et al., 2008). Thus, it is likely that more extensive work will reveal additional lincRNA mutants strains with additional brain and/or behavioral defects yet unexplored here. While rigorous behavioral studies will, in the future, determine whether loss of a lincRNA may result in specific behavioral abnormalities, we have made promising initial observations on two mutant strains. One mutant strain (*Spasm*) has tremors and a propensity to develop spastic movements upon handling, while a second strain (*linc–p21*) displays clasping of hind limbs when lifted by the tail (Sauvageau et al., unpublished).

As a whole, our 18 lincRNA knockout mouse models have revealed important aspects of lincRNA biology and constitute a useful resource for many future studies on the roles of lincRNAs in mammalian development, physiology and behavior.

## Materials and methods

### Mice

lincRNA knockout mice were generated by replacing the selected lincRNA gene with a *lacZ* cassette. Briefly, targeting constructs were constructed using VelociGene technology as described previously (Valenzuela et al., 2003). The VelociGene Allele Identification Numbers are shown in Supplementary file 1A. Linearized targeting constructs, generated by gap repair cloning containing mouse lincRNA upstream and downstream homology arms flanking a KOZAK-ATG-*lacZ*-pA-*LoxP*-hUb1-EM7-*neo*(superscript R)-pA-*LoxP* cassette, were electroporated into VGF1 hybrid mouse embryonic stem (ES) cells, derived from a 129S6S v/Ev female to a C57BL/6N male mating. Mouse ES cells carrying a heterozygous deletion of the lincRNA gene were identified by loss-of-function allele screening with 2 Taqman qPCR assays (Supplementary file 1F). Simultaneous replacement of the lincRNA gene with the *lacZ* cassette was confirmed by gain-of-allele Taqman assays against the *lacZ* and neomycin resistance cassette (Supplementary file 1F). Probes were labeled with 6-carboxy-fluorescein (FAM) on their 5′ ends and BHQ-1 on their 3′ ends. Targeted ES clones were introduced into an 8-cell stage mouse embryo using the VelociMouse method (Poueymirou et al., 2006). Mice were backcrossed once with C57BL/6J. Mutant mice were identified by genotyping for loss of lincRNA allele and gain of *lacZ* cassette. Toe clips, embryos or yolk sac were digested for 30 min at 95°C in 100 μl of 25 mM Sodium Hydroxide and 0.2 mM EDTA. Tissue digestion was neutralized by adding 100 μl of 40 mM Tris-HCl. PCR reactions using 4 μl of digested tissue with 10 mM *lacZ* specific and lincRNA gene specific primer pairs (Supplementary file 1C for sequences) were then performed and run on a 2% agarose gel. PCR conditions were as follows: 5 min at 95°C followed by 35 cycles of 30 s at 95°C, 45 s at 60°C, 30 s at 72°C and a final step at 72°C for 2 min. Mice were housed under controlled pathogen-free conditions (Harvard University’s Biological Research Infrastructure) and experiments were approved by the Harvard University Committee on the Use of Animals in Research and Teaching. Viability of the 18 lincRNA mutant strains was determined at postnatal day 21 by genotyping the progeny of heterozygous intercrosses (Supplementary file 1C for genotyping primer sequences). In the case of lethal strains, the developmental stage at which lethality occurs was determined by genotyping of embryos at E14.5 and E18.5 and newborns. Respiratory function (*Fendrr* mutant strain) was evaluated in surgically delivered E18.5 embryos from heterozygous intercrosses (Eggan et al., 2001). After cleaning of the airways, pups were placed on a 37°C warm pad and observed for sign of breathing.

### RNA isolation and mRNA-Seq library preparation

Total RNA from embryonic and postnatal mouse tissues, neural stem cells, and neurospheres was isolated using TRIzol (Life Technology, Carlsbad, CA)/chloroform extraction followed by spin-column purification (RNeasy mini kit, Qiagen, Venlo, Netherlands) according to the manufacturer instructions. RNA concentration and purity were determined using a Nanodrop (Thermo Fisher, Waltham, MA). RNA integrity was assessed on a Bioanalyzer (Agilent, Santa Clara, CA) using the RNA 6000 RNA chip. High-quality RNA samples (RNA Integrity Number ≥8) were used for library preparation. mRNA-seq libraries were constructed using the TruSeq RNA Sample Preparation Kit (Illumina, San Diego, CA) as previously described (Trapnell et al., 2012a; Sun et al., 2013). 500 ng total RNA was used as input for the TruSeq libraries from mouse tissues, and 200 ng for the libraries from neural stem cells and neurospheres. Prior to sequencing, libraries were run on a Bioanalyzer DNA7500 chip to assess purity, fragment size, and concentration. Libraries free of adapter dimers and with a peak region area (220–500 bp) ≥80% of the total area were sequenced. Individually barcoded samples were pooled and sequenced on the Illumina HiSeq 2000 platform.

### RNA-Seq analysis

Paired-end 101 bp reads were aligned to the mouse (mm9) reference genome assembly and, for the human neuronal differentiation time course also to the human (hg19) assembly, using Tophat2 (Trapnell et al., 2009) with default options and assembled into transcripts with Cufflinks (Trapnell et al., 2012a, 2012b). Aligned reads and assembled transcriptome catalog were used as input for Cuffdiff2 (Trapnell et al., 2012a) to determine expression levels (FPKM, Fragments Per Kilobase per Million mapped reads) and differential expression between conditions using default options. CummeRbund v2.1 (http://compbio.mit.edu/cummeRbund/) was then used to process, index, and visualize the output of the Cuffdiff2 analyses. Gene set enrichment analysis (GSEA) and Guilt-by-Association analysis (GBA) were performed to predict the effect of gene expression changes on biological processes. Detailed description of GSEA and GBA analysis, RNA isolation, and libraries preparation are provided in the Extended Experimental Procedures. *Cis*-enhancer activity was tested by determining the number of genes with differential expression in a particular Knockout vs wild type contrast within ±1 Mb window of the targeted lincRNA. 1000 random genomic intervals of the same size were obtained and interrogated in kind to determine how often the same number of differentially expressed (DE) genes could be identified. The ratio of intervals with DE genes >= the number of DE genes in the target-flanking window to the number of iterations, provided a bootstrapped p value and false discovery rate estimate.

### Gene set enrichment analysis (GSEA) and Guilt-by-Association (GBA) analysis

GSEA was performed to predict the effect of the significant gene expression changes on biological processes in the knockout mice. For a given comparison of KO vs WT differential expression, all genes were rank-ordered by their Cuffdiff2 test-statistics and mouse gene identifiers were mapped to human HUGO gene names. Genesets from the Reactome collection at mSigDB were obtained and for each gene set, the relative enrichment or depletion within our ranked list was determined via Mann-Whitney U-test. p values were corrected for multiple tests using the Benjamini-Hochberg method. Genesets with q<0.001 were selected and presented as a heatmap with color mapped to the Z-score of the Cuffdiff2 test statics for genes in the specific gene set relative to all genes. Redundant genesets were aggregated into higher-level biological processes.

Predictive GBA analysis for 17/18 tested lincRNAs was conducted as follows: Pearson correlation values of FPKM expression profiles were calculated for each lincRNA to all protein coding genes across a compendium of RNA-Seq samples (combination of in-house samples and samples from [Merkin et al., 2012]). Protein coding genes were then rank-ordered and subjected to the gene set enrichment analysis described above. Significant genesets for a given lincRNA represent the most likely pathways/biological processes for which this lincRNA may play a role.

### *lacZ* expression and histology

Expression of the knocked-in *lacZ* reporter gene was assessed in heterozygous mice. Embryos (from E13.5 to E18.5) were fixed in 4% paraformaldehyde (PFA) in phosphate buffered saline (PBS) overnight at 4°C prior to dissection of the brain, lung and respiratory tract, digestive tract, heart, and other organs. P7 brain, from *linc–Brn1b* mutant strain, were dissected from pups transcardially perfused with 4% paraformaldehyde (PFA), and fixed overnight at 4°C. The fixed tissues were rinsed three times at room temperature in PBS, 2 mM MgCl2, 0.01% deoxycholic acid, 0.02% NP-40. X-gal staining was performed by incubating the tissues for up to 16 hr at 37°C in the same buffer supplemented with 5 mM potassium ferricyanide, 5 mM potassium ferrocyanide and 1 mg/ml X-gal. Staining reaction was stopped by washing three times in PBS at room temperature, followed by 2 hr post-fixation in 4% PFA at 4°C. Stained whole organs and sagittal brain sections were imaged using a Leica M216FA stereomicroscope (Leica Microsystems, Buffalo Grove, IL) equipped with a DFC300 FX digital imaging camera (Watson et al., 2008)*.* Histology was performed at the Rodent Histopathology Service of the Dana Farber/Harvard Cancer Center Pathology Research Core. Embryos were harvested, fixed in Bouin’s solution and embebbed in paraffin. Microtome sections were stained with hematoxilin and/or eosin for histological analysis.

### Nissl staining and immunocytochemistry

Embryonic brains, dissected in cold PBS and fixed in 4% PFA/PBS overnight at 4°C, and P7 brains dissected from pups transcardially perfused with 4% PFA and post-fixed as described above, were processed for Nissl staining and immunofluorescence as previously described (Englund et al., 2005; Molyneaux et al., 2005)*.* Nissl-stained and immunostained sections were imaged using a Nikon 90i fluorescence microscope equipped with a Retiga Exi camera (Q-IMAGING, Surrey, Canada) and acquired with Volocity image analysis software v4.0.1 (Perkin Elmer, Waltham, MA). For quantification of overall cortical thickness, cortical layers and number of CUX1+, CTIP2+ and TLE4+ cells within the primary somatosensory cortical area, anatomically matched sections were processed (n = 3 *linc–Brn1b*^−*/*−^; n = 3 wild-type, at P7). Boxes of 300 pixels in width and spanning the thickness of the cortex were superimposed at matched locations on each section, and the overall cortical thickness was measured as the distance from the *pia* to the white matter in each box, using ImageJ. Specific layer thicknesses were measured at the midpoint of the matched-location 300 pixel images for each of the TLE4^+^, CTIP2^+^ and SATB2^+^ immunofluorescence stainings using ImageJ. Layer VI thickness was measured as the distance between the dorsal edge of the TLE4^+^ region and the white matter. Layer V thickness was determined by the span of the CTIP2^+^ region, and layer II–IV thickness were measured as the SATB2^+^ region between the dorsal edge of the CTIP2^+^ stain and the pia. In each case results were expressed as mean ± SEM. Cell counts of the specific neuronal subpopulations were obtained using the ITCN plugin for ImageJ and results were expressed as mean ± SEM. A priori criteria were defined for analysis. Statistical analysis was performed using R unpaired Student’s *t* test assuming equal variance was used for the pairwise comparisons.

### Antibodies and image acquisition

Primary antibodies and dilutions were as follows: anti-ßgal (CGAL-45; 1:500; Immunology Consultants Laboratory, Portland OR), anti-SATB2 (ab51502; 1:50; Abcam, Cambridge, UK), anti-CUX1 (sc-13024; 1:100; Santa Cruz Biotechnology, Dallas, TX), anti-pH3 (06-570; 1:500; Millipore, Billerica, MA), anti-TBR2 (1:2000; Gift from Robert F Hevner Lab), anti-CTIP2 (ab18465; 1:100; Abcam), anti-TLE4 (sc-9125; 1:100; Santa Cruz), anti-vGLUT2 (MAB5504; 1:50; Millipore), anti-5-HTT (PC177L; 1:1000; Millipore), anti-TUJ1 (mms-435P; 1:1000; Covance, Princeton, NJ). Secondary antibodies were from the Molecular Probes (Eugene, OR) Alexa series and were used at 1:750 dilution. Immunostained sections were imaged using a Nikon 90i fluorescence microscope equipped with a Retiga Exi camera (Q-IMAGING) and acquired with Volocity image analysis software v4.0.1 (Improvision).

### Cytochrome oxidase staining

*linc–Brn1b*^−/−^ and wild-type littermates at P7 were anesthetized, perfused with 4% PFA, decapitated, and the brain rapidly removed. The brains were post-fixed in 4% PFA for 3 hr at 4°C. Cortices containing the barrel field were dissected and flattened as described (Welker and Woolsey, 1974), post-fixed in 4% PFA for 12–14 hr at 4°C and sectioned by using a vibratome (80 μm). Sections were incubated in phosphate buffer containing 0.5 mg/ml DAB, 0.18 mg cytochrome C, and 40 mg/ml sucrose for 3–5 hr at 37°C, rinsed, and mounted in Fluoromount-G (SouthernBiotech, Birmingham, AL) (Wong-Riley, 1979; Land and Simons, 1985).

### In situ hybridization

Nonradioactive in situ hybridization was performed on 40 μm vibratome sections mounted on superfrost slides (Fisher Scientific, Waltham, MA) as previously described (Berger and Hediger, 2001; Arlotta et al., 2005). The probe for *Svet1* was a gift from M Studer. Probes for *RorB* (nt 1573-2087 of NM_146095) and *Cux2* (nt 1069-11694 of NM_007804) transcripts were generated by PCR from mouse brain cDNA and subcloned in pCRII-TOPO (Life Technologies, Carlsbad, CA). Antisense riboprobes were generated by *in vitro* transcription using SP6 or T7 polymerase (Roche Applied Science, Penzberg, Germany) as previously described. Sense probes were used as negative controls.

### Single molecule FISH

Single molecule FISH was performedas described by Raj et al. (2008). Briefly, oligonucleotide probes targeting and tiling *Peril* (48 probes) and *linc–Brn1b* (20 probes) were conjugated to Quasar 570 fluorophores and HPLC purified (Biosearch Technologies, Petaluma, CA). A list of the *Peril* and *linc–Brn1b* probes (sequence, position, and %CG content) is provided in Supplementary file 1E. Dissociated E14.5 cortical neurospheres or mouse ES cells were fixed in 2% formaldehyde for 10 min, washed twice with PBS, and permeabilized with 70% ethanol. The cells were then seeded onto previously gelatinized two-chamber cover glasses. Prior to hybridization, the cells were rehydrated in wash buffer containing 10% formamide and 2 × SSC for 5 min. Probes (0.5 ng/μl final concentration) were hybridized in 10% dextran sulfate, 10% formamide, and 2 × SSC at 37°C overnight. After hybridization, cells were washed twice with wash buffer at 37°C for 30 min (with DAPI added to the second wash for nuclear staining), and twice with 2 × SSC. After the SSC wash, the cells were equilibrated in anti-fade buffer (2 × SSC, 0.4% glucose, 10 mM Tris pH 8.0) for 3–5 min. Cells were mounted in 100 μl anti-fade buffer supplemented with 1 μl of glucose oxidase (G2133-10KU; Sigma-Aldrich, St. Louis, MO) and 1 μl of catalase (C3515-10 MG; Sigma-Aldrich) and immediately imaged with a LSM 700 Inverted Confocal microscope (Zeiss, Jena, Germany). 25 Z-stacks were taken per field, using DAPI and laser 639 for excitation.

### Human neural stem cell culture and differentiation

H1 human neural stem cells were prepared as described previously (Arnold et al., 2008; Goff et al., 2009) and grown at 37°C, 5% CO_2_ on 1:4 diluted Matrigel-coated wells in neural proliferation medium (NPM; 50% DMEM/F12 Glutamax, 50% Neurobasal medium, 0.5X N2, 0.5X B27 without vitamin A, 20 ng/ml FGF [Life Technologies]). For differentiation, cells were plated at a density of 10^6^ cells per well in a 6-well plate and allowed to proliferate for one day in the NPM medium. Neural induction was then initiated by withdrawal of FGF and addition of BDNF by switching the medium to neural differentiation medium (NDM; 100% Neurobasal medium, 1X B27 without vitamin A [Life Technologies], 10 ng/ml BDNF [Peprotech, Rocky Hill, NJ].) Differentiating cultures were maintained by refreshing NDM every other day until collection. Samples of these cultures were collected at days 0, 1, 2, and 4. Remaining cells (those designated for collection at days 5, 11, and 18) were replated at day 4 at a density 10^6^ cells per well of a Poly-D-lysine/laminin-coated 6-well plate. Cells were harvested with Accutase (Stem Cell Technologies, Vancouver, Canada) and RNA collected as described above.

### Western blot

*linc–Brn1b*^*−/−*^ and WT E14.5-derived neurospheres (passage 3) and E15.5 cortices were lysed in RIPA buffer (1% NP-40, 1% Na-deoxycholate, 0.2% SDS, 50 mM Tris 7.4, 500 mM NaCl) containing protease inhibitor cocktail (Roche Applied Science). Proteins were resolved by SDS-PAGE and electroblotted. Blots were probed sequentially for BRN1 (anti-BRN1, sc-6028-R, Santa Cruz) and GAPDH (anti-GAPDH, sc-365062-R, Santa Cruz) to control for protein loading. Immunoreactive bands were detected by enhanced chemiluminescence (SuperSignal, Thermo Fisher Scientific, Waltham, MA), and visualized with a Gel Doc (Biorad).

### Accession numbers

All primary RNA-Seq data are available at the Gene Expression Omnibus (GSE49581).

## Additional files

Supplementary file 1. (**A**) lincRNA genomic and targeted deletion coordinates (mm9). (**B**) Mass spectrometry peptide tags at background level identified for 4 lincRNA candidates (lif-over of lincRNA candidates to hg19). (**C**) Mouse genotyping primer sequences. (**D**) Quantitative RT-PCR primers. (**E**) Single molecule FISH probe sequences. (**F**) Taqman qPCR primers for targeted mES diagnostic. **DOI:** http://dx.doi.org/10.7554/eLife.01749.018

### Major datasets

The following dataset was generated:

SauvageauM, GoffLA, LodatoS, BonevB, GroffAF, GerhardingerC, , 2013, Multiple knockout mouse models reveal lincRNAs are required for life and brain Development, ; http://www.ncbi.nlm.nih.gov/geo/query/acc.cgi?acc=GSE49581, Publicly available at NCBI GEO (http://www.ncbi.nlm.nih.gov/geo/).

The following previously published datasets were used:

MerkinJ, RussellC, ChenP, BurgeCB, 2012, Evolutionary dynamics of gene and isoform regulation in mammalian tissues, ; http://www.ncbi.nlm.nih.gov/geo/query/acc.cgi?acc=GSE41637, Publicly available at NCBI GEO (http://www.ncbi.nlm.nih.gov/geo/).

IngoliaNT, LareauLF, WeissmanJS, 2011, Ribosome profiling of mouse embryonic stem cells reveals the complexity of mammalian proteomes, ; http://www.ncbi.nlm.nih.gov/geo/query/acc.cgi?acc=GSE30839, Publicly available at NCBI GEO (http://www.ncbi.nlm.nih.gov/geo/).

ShenY, YueF, McClearyDF, YeZ, EdsallL, KuanS, , 2012, A draft map of cis-regulatory sequences in the mouse genome, ; http://www.ncbi.nlm.nih.gov/geo/query/acc.cgi?acc=GSE29184, Publicly available at NCBI GEO (http://www.ncbi.nlm.nih.gov/geo/).
